# Direct Evidence for Pitavastatin Induced Chromatin Structure Change in the *KLF4* Gene in Endothelial Cells

**DOI:** 10.1371/journal.pone.0096005

**Published:** 2014-05-05

**Authors:** Takashi Maejima, Tsuyoshi Inoue, Yasuharu Kanki, Takahide Kohro, Guoliang Li, Yoshihiro Ohta, Hiroshi Kimura, Mika Kobayashi, Akashi Taguchi, Shuichi Tsutsumi, Hiroko Iwanari, Shogo Yamamoto, Hirofumi Aruga, Shoulian Dong, Junko F. Stevens, Huay Mei Poh, Kazuki Yamamoto, Takeshi Kawamura, Imari Mimura, Jun-ichi Suehiro, Akira Sugiyama, Kiyomi Kaneki, Haruki Shibata, Yasunobu Yoshinaka, Takeshi Doi, Akimune Asanuma, Sohei Tanabe, Toshiya Tanaka, Takashi Minami, Takao Hamakubo, Juro Sakai, Naohito Nozaki, Hiroyuki Aburatani, Masaomi Nangaku, Xiaoan Ruan, Hideyuki Tanabe, Yijun Ruan, Sigeo Ihara, Akira Endo, Tatsuhiko Kodama, Youichiro Wada

**Affiliations:** 1 Research Center for Advanced Science and Technology, The University of Tokyo, Meguro-ku, Tokyo, Japan; 2 Tokyo New Drug Research Laboratories, Kowa Company Ltd., Higashimurayama, Tokyo, Japan; 3 Division of Nephrology and Endocrinology, The University of Tokyo School of Medicine, Bunkyo-ku, Tokyo, Japan; 4 Department of Translational Research for Healthcare and Clinical Science, Graduate School of Medicine, The University of Tokyo, Bunkyo-ku, Tokyo, Japan; 5 Jackson Laboratory for Genomic Medicine, Farmington, Connecticut, United States of America; 6 Graduate School of Frontier Biosciences, Osaka University, Suita, Osaka, Japan; 7 Thermo Fisher Scientific, South San Francisco, California, United States of America; 8 Mab Institute Inc., North Advancement Center for Science & Technology, Sapporo, Hokkaido, Japan; 9 Genome Institute of Singapore, Singapore, Singapore; 10 Department of Evolutionary Studies of Biosystems, School of Advanced Sciences, The Graduate University for Advanced Studies (Sokendai), Hayama, Kanagawa, Japan; 11 Biopharm Research Laboratories, Inc., Koganei, Tokyo, Japan; 12 Radioisotope Center, The University of Tokyo, Bunkyo-ku, Tokyo, Japan; Brigham and Women's Hospital, Harvard Medical School, United States of America

## Abstract

Statins exert atheroprotective effects through the induction of specific transcriptional factors in multiple organs. In endothelial cells, statin-dependent atheroprotective gene up-regulation is mediated by Kruppel-like factor (*KLF*) family transcription factors. To dissect the mechanism of gene regulation, we sought to determine molecular targets by performing microarray analyses of human umbilical vein endothelial cells (HUVECs) treated with pitavastatin, and *KLF4* was determined to be the most highly induced gene. In addition, it was revealed that the atheroprotective genes induced with pitavastatin, such as nitric oxide synthase 3 (*NOS3*) and thrombomodulin (*THBD*), were suppressed by *KLF4* knockdown. Myocyte enhancer factor-2 (*MEF2*) family activation is reported to be involved in pitavastatin-dependent *KLF4* induction. We focused on *MEF2C* among the *MEF2* family members and identified a novel functional *MEF2C* binding site 148 kb upstream of the *KLF4* gene by chromatin immunoprecipitation along with deep sequencing (ChIP-seq) followed by luciferase assay. By applying whole genome and quantitative chromatin conformation analysis {chromatin interaction analysis with paired end tag sequencing (ChIA-PET), and real time chromosome conformation capture (3C) assay}, we observed that the *MEF2C*-bound enhancer and transcription start site (TSS) of *KLF4* came into closer spatial proximity by pitavastatin treatment. 3D-Fluorescence in situ hybridization (FISH) imaging supported the conformational change in individual cells. Taken together, dynamic chromatin conformation change was shown to mediate pitavastatin-responsive gene induction in endothelial cells.

## Introduction

Since the discovery of the 3-hydroxy-3-methylglutaryl coenzyme A reductase inhibitors [1], commonly known as statins, they have come to be widely-used cholesterol lowering drugs [2], [3]. Multiple lines of evidence, including randomized clinical trials, have suggested that statins also directly affect vascular cells, and exert atheroprotective effects through a modification of gene expression [4]. In endothelial cells (ECs), statins induce the mRNA level of nitric oxide synthase 3 *(NOS3)* and thrombomodulin *(THBD)*, and this effect is reported to be mediated by the induction of the transcription factor krüppel-like factor 2 *(KLF2)* acting through the myocyte enhancer factor 2 *(MEF2)* binding site [5], [6]. Small-interfering RNA (siRNA)-mediated knockdown of *KLF2* strongly attenuates the ability of statins to increase *NOS3* and *THBD* accumulation in ECs. Thus, statins are thought to exert their atheroprotective effects through *KLF2* to some extent.

Krüppel-like factors are zinc finger transcriptional factors that have been implicated in blood vessel development and T lymphocyte activation [7]. Previous studies suggested that *KLF2* functions as a regulator of inflammation, and also participates in vasodilatation and anti-coagulation [8], [9]. In the context of ECs subjected to shear stress, *KLF2* reportedly mediates the expression of a series of response genes [10], [11]. *KLF2* is of particular interest in atherogenesis, because cholesterol accumulation and low shear stress in the vascular wall are two major aspects of atherosclerotic plaque formation, suggesting that the *KLF* family exerts important biological effects on the cellular phenotype. Thus, we focused on *KLFs* to investigate the molecular mechanism of gene regulation in statin-treated vascular cells.

Human umbilical vein endothelial cells (HUVECs) are primary cultivated endothelial cells widely used in vascular biology research and provided a critical model for molecular mechanism of atherosclerosis, because umbilical vein carries oxygenated blood flow just as arteries [4], [9], [12], [13]. To validate the contribution of *KLFs* in HUVECs under statin treatment, we performed transcriptome analysis using a microarray and statistically identified the affected genes. Unexpectedly, *KLF4* was induced more than the others, including *KLF2*. Since the importance of *KLF4* in the regulation of other atheroprotective genes was thus suggested, we focussed on the molecular mechanisms of pitavastatin-dependent *KLF4* induction.

## Results

### Pitavastatin induces atheroprotective genes through *KLF4* in both HUVECs and the aortic endothelium of ApoE deficient mice

**Figure 1:**
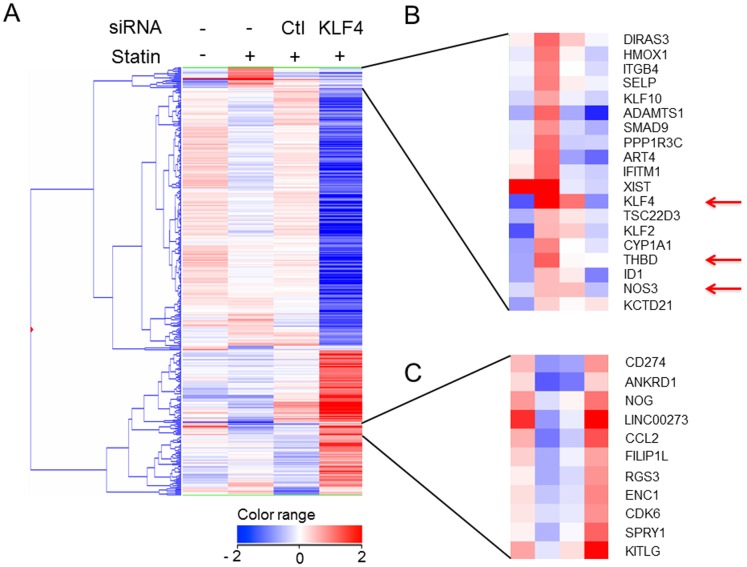
Genes up- or down-regulated by pitavastatin treatment through *KLF4* in HUVECs. Transcriptome data were derived from the average of an array performed 5 times with 1 µM pitavastatin-treated HUVECs and the average of duplicate arrays using HUVECs transfected with *KLF4* siRNA or control (Ctl) siRNA, and treated with 1 µM pitavastatin for 4 hours. Fold induction is the representation of a log2 fold change to standardize the induction rate. Whole clustering analysis (A) using 384 selected genes that had significant changes in expression compared to control treatment were selected (See the details in *Methods*). The cluster shown in (B) contains the genes induced by pitavastatin and suppressed with si*KLF4*. Note that *NOS3* and *THBD* are included in addition to *KLF4*. These genes are indicated with red arrows. *KLF2* is shown by black arrow. The cluster shown in (C) includes the genes reduced pitavastatin treatment and induced with si*KLF4*. The sequences of the applied siRNA are shown in Table S2A in File S1.

First, to evaluate the gene expression profiles of the KLF family members, we performed microarray analysis of HUVECs treated with pitavastatin for 4 hours. Based on the results of repeated experiments, the most highly induced gene was *KLF4*, followed by *KLF2* (Table S1 in File S1). To confirm that *KLF4* induction participates in statin-dependent atheroprotective gene induction, we performed further microarray analyses with a siRNA against *KLF4* following the procedure shown in Figure S1 in File S1. The changes in gene expression in pitavastatin treatment/DMSO treatment and si*KLF4*/siControl under pitavastatin were calculated. The genes that exhibited significant changes (fold change> = 2.0 or < = 0.5) in either condition were selected for further study (384 genes). Hierarchical clustering analysis was performed after selecting the genes (Figure 1*A*). Figure 1*B* shows the cluster of genes induced by pitavastatin treatment and suppressed by si*KLF4*. In this cluster, 17 other genes were classified in addition to *KLF4* and *KLF2*, among which *NOS3* and *THBD* were present (Fig. 1*B*). This result showed the statin-dependent induction of these atheroprotective genes, an effect which was suppressed by *KLF4* knockdown, suggesting *KLF4* functions as a key regulator of these genes in HUVECs. Figure 1C shows the cluster that includes genes having the opposite expression pattern to that in Figure 1B, i.e. suppressed by pitavastatin and enhanced by si*KLF4*. An inflammatory mediator, chemokine (C-C motif) ligand 2 (*CCL2*) was also included in this cluster.

To further investigate the correlation between *KLF4* and *THBD* or *NOS3*, we performed quantitative real time PCR to determine the time course profile of mRNA expression. A significant induction of *KLF4* was observable within 2 hours of pitavastatin treatment, followed by *THBD* and *NOS3* mRNA induction after 4 hours (Fig. S2*A* in File S1).

To test whether the induction of these genes by statins also occurred *in vivo*, we collected mRNA after 12-week pitavastatin treatment from the aortic tissue of ApoE deficient mice that had developed advanced atherosclerotic lesions. As shown in Figure S2*B* in File S1, the mRNA of *Klf4, Thbd, and Nos3* was induced by pitavastatin. Histological analysis furthermore showed that pitavastatin treatment reduced the plaque (Fig. S3*A* in File S1) in spite of the fact that the total cholesterol and triglyceride levels were not significantly affected (Fig. S3*B* in File S1). Taking these several lines of *in vitro* and *in vivo* evidence together, it appears that *KLF4* was indispensable for the endothelial transcriptional activation induced by statins.

### Whole genome profile of the *MEF2C* binding sites in HUVECs

To dissect the molecular mechanism of transcriptional regulation of *KLF4*, we set out to identify the key transcription factors involved. In the case of *KLF2*, there are reports of a *MEF2* binding site in the *KLF2* promoter [5], [11], but in the *KLF4* promoter region, the location has not still been identified. Therefore, we made new antibodies against *MEF2A*, *MEF2C*, *KLF2* and *KLF4* (Fig. S4 in File S1). Only by knocking down *MEF2A*, *C* and *D* was a reduction of both *KLF2* and *KLF4* achieved, presumably due to functional redundancy (Fig. S5 in File S1). However, based on our transcriptome database on HUVECs (http://sbmdb.genome.rcast.u-tokyo.ac.jp/refexa/advanced_search.jsp), we focused on *MEF2C*, because it is expressed more potently than the two other *MEF2* family members in HUVECs. To identify the transcription regulatory elements by which *MEF2C* contributes to the *KLF4* induction under pitavastatin treatment, we made an effort to identify the binding sites in a whole genome manner. A newly established monoclonal antibody against *MEF2C* (Fig. S4*D* in File S1) was used to perform chromatin immunoprecipitation followed by deep sequencing (ChIP-seq). As shown in Figure S6*A* in File S1, 4,878 binding sites were detected in the control [pitavastatin (−)], with 42% of the *MEF2C* binding sites located between the TSS and the 3′UTR of the genes, while the remaining 58% of the *MEF2C* binding sites were in intergenic regions. Among all of the *MEF2C* binding sites, 16.4% co-localized with H3K27ac (Figure S6*B* in File S1), which is suggestive of an enhancer [14]. In contrast, 13,030 binding sites were found in the statin-treated HUVECs, with 40% of the *MEF2C* binding sites between the TSS and the 3′UTR, 60% in intergenic regions (Figure S6*C* in File S1). There was no evident significant difference in the *MEF2C* binding distribution profile between the untreated and statin-treated HUVECs.

### Statin-mediated induction of *KLF4* involves the binding of *MEF2C* to a distal kb –148 enhancer

**Figure 2:**
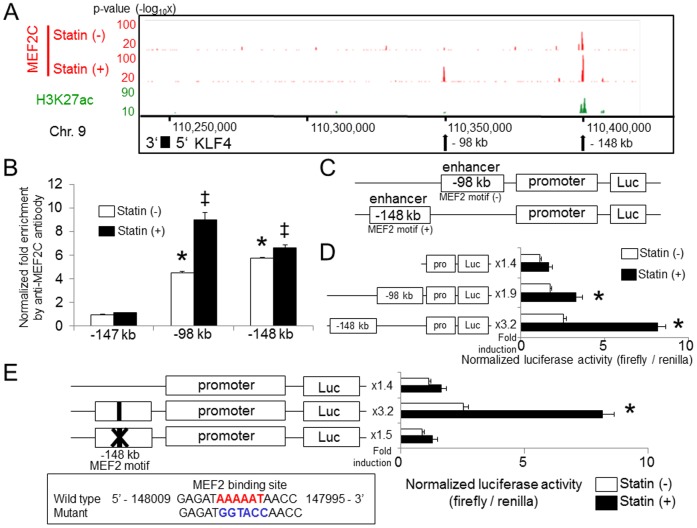
Binding of *MEF2C* at kb −148 from the TSS of the *KLF4* gene is essential to pitavastatin-mediated *KLF4* induction. (A) HUVECs were incubated with 1 µM pitavastatin for 4 hours. As described in *Methods*, Chromatin immunoprecipitation was performed followed by deep sequencing. The localization and magnitude of *MEF2C* binding in the *KLF4* transcription regulation region are illustrated. Two *MEF2C* binding sites in the *KLF4* locus (−98 and −148 kb, relative to the TSS) were detected by ChIP-seq analysis. The localization of H3K27ac obtained by ChIP-seq is shown in the third lane. (B) Schematic structure of the transcriptional regulation region of the *KLF4* gene. The sequences of the primers used are shown in Table S2D in File S1. (C) HUVECs were transiently transfected with a KLF4-luc, (−98 kb)-KLF4-luc and (−148 kb)-KLF4-luc plasmid together with the *Renilla* luciferase plasmid, and were treated with 1 µM pitavastatin for 12 hours. Luciferase activity was measured as described in the *Methods* section. Error bars indicate the S.D. (*n* = 3), *P<0.01 compared with pitavastatin (−), Student's t test. (D) HUVECs were transiently transfected with KLF4-luc, wild-type enhancer (−148 kb)-KLF4-luc and (enhancer −148 kb)-KLF4-luc containing a point mutation in the *MEF2* binding element. Pitavastatin-mediated induction of promoter activity was abolished by mutation of the *MEF2C* binding site. Error bars indicate the S.D. (*n* = 3), *P<0.01 compared with pitavastatin (−), Student's t test. The *Firefly* luciferase activity value was normalized by *Renilla* luciferase activity.

In the *KLF4* locus, two *MEF2C* binding regions were detected after statin treatment (kb −98 and kb −148, relative to the TSS) (Fig. 2*A*). Sustained *MEF2C* binding was specifically enriched in the kb −148 kb region. Increased *MEF2C* binding at the two loci was also qualified by ChIP-qPCR (Fig.S7 in File S1). Histone modification of H3K27ac was observable in the −98 kb region, but was more clearly evident in the −148 kb region (Fig. 2*A*).

To determine which *MEF2C* binding sites are functionally active, we carried out a series of luciferase reporter assays in HUVECs with constructs containing the human *KLF4* promoter (between bp −994 and +594 bp) coupled to the *MEF2C* binding region from kb −98 (255 bp), or kb −148 (506 bp) (Fig. 2*B*). As shown in Figure 2*C*, pitavastatin exhibited a 1.4 fold induction of the *KLF4* promoter construct, and once coupled to the kb −98 *MEF2C* binding region, a 1.9 fold induction was exhibited. Using the construct containing both the promoter and the kb −148 *MEF2C* binding region that was induced by pitavastatin, a more potent induction of 3.2 fold was observed (Fig. 2*C*), suggesting that the kb −148 *MEF2C* binding region is the more important one in pitavastatin-dependent *KLF4* induction.

By consensus motif search, we found one *MEF2* binding motif in the kb −148 enhancer region. Based on this sequence, we generated the enhancer (−148 kb)-*KLF4*-luc containing a 6 base mutation (GAGATAAAAATAACC to GAGATGGTACCAACC) of the *MEF2* binding site to determine the importance of this motif for statin-mediated *KLF4* promoter activation. As shown in Figure 2*D*, the statin-mediated induction of promoter activity was abolished by a mutation in the *MEF2C* binding site. Taking these results together, statins increased the expression of *KLF4* in ECs by activating the *MEF2C* binding element in the −148 kb region.

### The distal kb −148 enhancer in the *KLF4* gene directly interacts with the promoter with the frequency of their interactions increased by statins

To elucidate how this kb −148 enhancer of *KLF4* gene functions, we sought to determine the localization of transcriptionally active RNA polymerase II (Pol II). To perform ChIP-seq, a monoclonal antibody was established against the C-terminal domain (CTD) of the largest subunit of Pol II, in which the tetramer repeats (Y_1_S_2_P_3_T_4_S_5_P_6_S_7_) contain two phosphorylation sites at the second and fifth Serine (Methods, Fig. S8 in File S1). Since this phosphorylation pattern of the CTD is regarded as a mark of elongating Pol II, the localization of the binding signal at both the TSS of *KLF4* and the −148 kb site suggested the possibility that the two sites might be in spatial proximity of one another.

**Figure 3:**
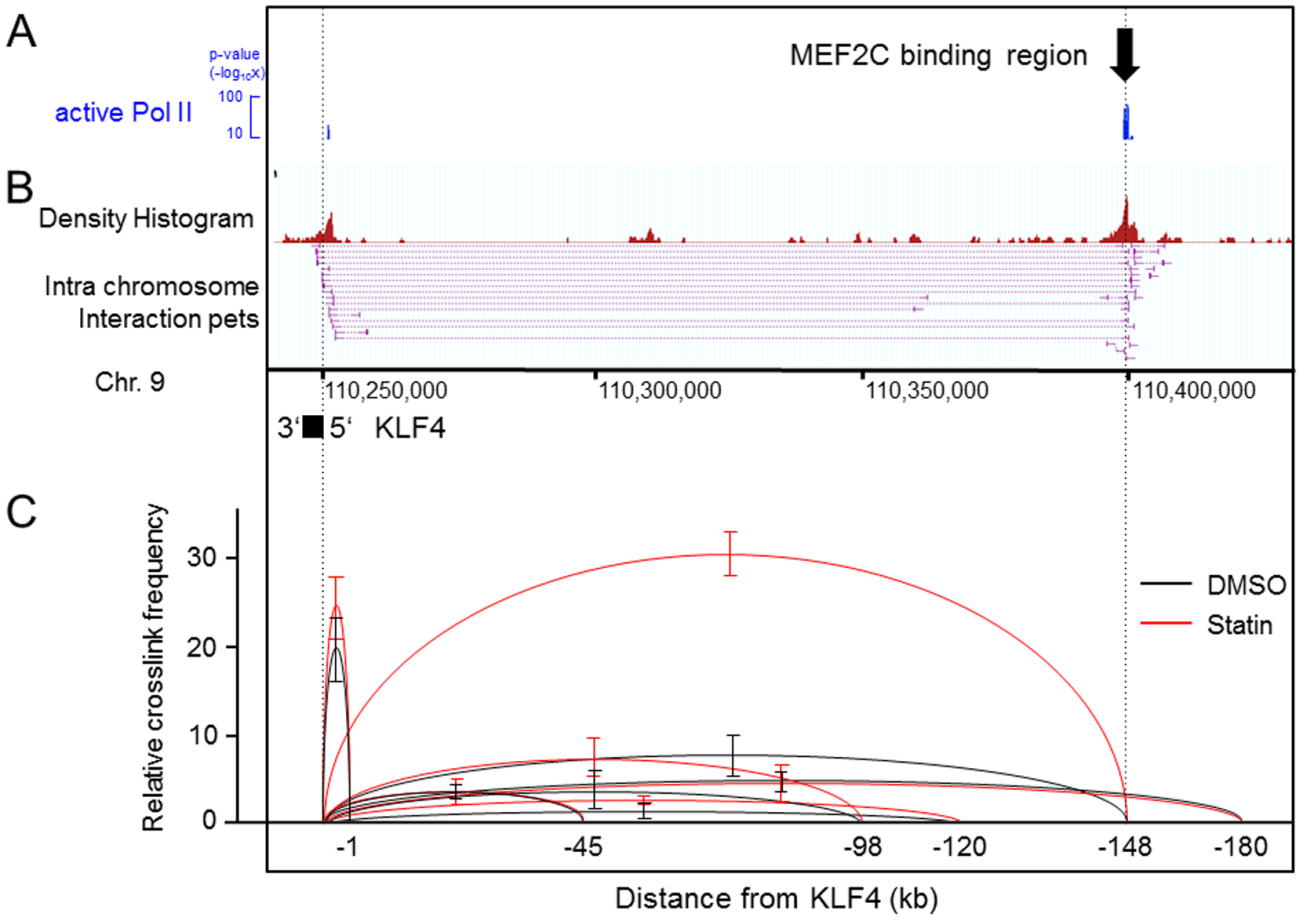
The frequency of direct interaction between the kb −148 enhancer and promoter in the *KLF4* locus was affected by pitavastatin treatment. HUVECs were harvested and cultivated as described in the *Methods* section. (A) The localization of active Pol II obtained by ChIP-seq. The black arrow shows the *MEF2C* binding site identified by ChIP-seq. (B) A ChIA-PET library was constructed and sequenced. From the TSS of *KLF4*, 15 PETs originated and 13 of them interacted with a locus −148 kb upstream of the TSS, which result is identical with the *MEF2C* binding site observed by ChIP-seq and validated by luciferase assay in Figure 2. The numbers in the middle indicate the location on chromosome 9 using the hg19 build program. (C) Quantitative 3C assay. HUVECs were incubated with 1 µM pitavastatin for 4 hours. Primers were designed for analyzing the crosslink frequency of the regions connected with the arches. The relative frequencies were compared between DMSO control (black arch) and statin treatment (red arch). The sequences of the primers are shown in Table S2E in File S1. The data (mean ± SD) is representative of three independent experiments with similar results. Note that the interaction between the TSS and kb −148 was increased by statin treatment.

As was reported previously [15], a target gene promoter is able to come into a conformational proximity with an active enhancer, and the frequency with which this takes place is related to transcriptional activity. To test whether this gain of interaction between the newly identified enhancer −148 kb upstream of *KLF4* and the promoter region contributes to *KLF4* induction by statins, and to evaluate the relative conformational distance, we performed two different kinds of chromosome conformation capture (3C) based experiments. First, to test whether the distal enhancer directly communicates with the promoter region, we performed whole genome chromatin interaction analysis with paired end tag sequencing (ChIA-PET) [16] using the same elongating Pol II antibody shown in Figure 3*A*. As shown in Figure 3*B*, direct interaction between the −148 element and the promoter region of *KLF4* was detected in the serum-starved control, suggesting this enhancer may co-exist with the promoter in the Pol II rich transcription complex [17].

Next, to evaluate the effect of statins on the frequency of spatial proximity between the kb −148 enhancer and promoter, we performed quantitative 3C experiments using TaqMan PCR primers (Methods and Table S2E in File S1). In the DMSO treated control, the −148 kb distal element exhibited a higher frequency than the other regions but a lower frequency than the −1 kb region (Fig. 3*C*, lower column, black arch). However, after statin treatment, the frequency of spatial proximity between kb −148 and TSS increased a further 5 fold (Fig. 3*C*, lower column, red arch). To validate the specificity of the assay, the generated PCR products were all sequenced, and the direct connection between the TSS of *KLF4* and the −148 kb element was confirmed (Fig. S9 in File S1). Considering these findings together, the *KLF4* gene locus takes a conformation in which the kb −148 enhancer and promoter are in close spatial proximity by virtue of Pol II. After statin treatment, the distal enhancer and promoter come to be situated close to each other more frequently due to changes in chromatin conformation caused by the recruitment of more *MEF2C* to the *KLF4* locus.

### 3D-FISH confirms the proximity of the *KLF4* and MEF2C binding region that was detected by 3C

**Figure 4:**
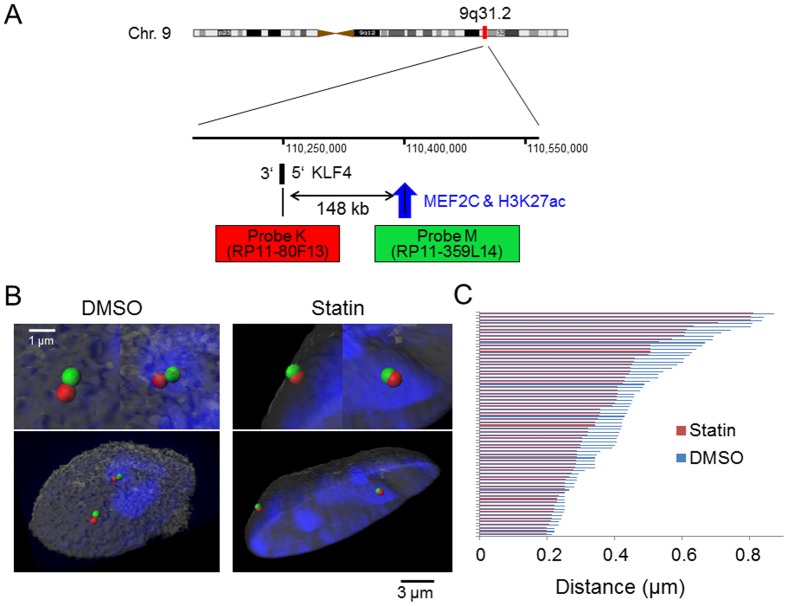
3D-FISH confirms the proximity of *KLF4* and the *MEF2C* binding region detected by 3C. HUVECs were incubated with 1 µM pitavastatin for 4 hours. (A) Probe design for the two-color 3D-FISH analysis of the target region on human chromosome 9q31.2. The numbers in the middle indicate the location on chromosome 9 using the hg19 build program. (B) Visualization of two-color 3D-FISH on structurally preserved HUVEC nuclei and an image of the 3D distance. FISH with probes K (red) and M (green) showing the *KLF4* gene and *MEF2C* binding region, respectively. Nuclei were counterstained with TOPRO-3 (blue). 3D reconstruction was carried out on the captured image with Imaris software. The left panel shows the representative image of HUVECs with DMSO and the right panel shows the representative image of HUVECs with statin treatment. Magnified views of each probe sets are shown on top of the whole images. (C) The distance between the *KLF4* gene and *MEF2C* binding region for each condition. The distance was measured using the 3D image processing and analysis software CTMS (Chromosome Territory Measurement Software) (Cybernet Co. Ltd.). 70 chromosomes were analyzed and all of the data are shown in this figure. The average distances between the *KLF4* gene and *MEF2C* binding region are 0.45 µm with DMSO and 0.38 µm with the statin. *P*<0.05 compared with pitavastatin (−), Wilcoxon rank-sum test.

To validate chromatin conformation change over a span of 148 kb at the single cell level, two-color-3D-FISH experiments were performed. As shown in Figure 4*A*, probes for the *KLF4* gene and the kb −148 *MEF2C* binding region were used to visualize the two regions. The two probes were validated, as shown in Figure S10 in File S1. Figure 4*B* indicates the representative images of 3D-FISH under the two conditions. In DMSO-treated cells, the TSS and *MEF2C* bound −148 kb site were distinguished as two foci, but after statin treatment, two different kinds of foci were apparently merged together, indicating that they are in spatial proximity. In addition to these representational images, we collected images from more than 70 nuclei in each condition and statistically analyzed the 3D intergenic distance between the two target regions to determine whether the proximity changed with pitavastatin. As shown in Figure 4*C*, the distance between TSS and kb −148 became closer after statin treatment. This result visually reinforced the finding of chromatin conformational change caused by pitavastatin that was detected using the 3C assay actually took place on an individual cell basis.

## Discussion

HMG-CoA reductase inhibitors (statins) exert certain beneficial effects, especially a reduction of the plasma cholesterol levels. Various reports have also suggested that direct effect on vascular wall activity might also contribute to the atheroprotectiveness of the statins [4]. In this report, we focused on endothelial cells and performed comprehensive gene expression analysis using pitavastatin, one of the clinically used statins [3]. As shown in Figure 1*A*, the transcription factor, *KLF4* was the most highly induced gene.

*KLF* family members are reported to exert anti-atherogenic effects in both *in vivo* and *in vitro* models [18] [19]. Among the genes induced (Figure 1*B*), certain atheroprotective genes, including *NOS3, THBD* and a disintegrin and metalloproteinase with thrombospondin motif 1 (*ADAMTS1*), were identified [20]. In addition, the genes specifically suppressed by pitavastatin were also identified and the most reduced gene was the inflammatory mediator chemokine (C–C motif) ligand 2 (*CCL2*) (Figure 1*C*). These results are in accord with the previous notion that *KLF4* is beneficial to vascular cells due to both an induction of anti-atherogenic genes and suppression of inflammatory mediators [19].

The signaling cascade elicited by statin treatment that exerts an effect on *KLF4* induction is not well understood, although a few critical pathways have been reported [6], [11], [21], [22]. The *MEK5/ERK5/MEF2C* pathway was shown to participate in the maintenance of the vascular network [23], [24], [25] and some of the regulatory elements in the induction of *KLF4* have been reported [5], [11]. In general, and as was reported in HeLa cells [26], three of four *MEF2* family members, *MEF2A*, -*2C* and -*2D*, are expressed in ECs. While there was no vascular phenotype reported for *MEF2D*-deficient mice [27], the reported vascular insufficiency in *MEF2C*-deficient mice suggests an essential role for *MEF2C* [25]. A mutation of *MEF2A* was also reported in an inherited disorder with features of coronary artery disease [28], suggesting its participation in endothelial function. While the importance of *MEF2C* was demonstrated in this report, the relative importance and the interactions of the *MEF2* family members involved in statin-dependent *KLF4* induction await further elucidation.

*MEF2C* binding sites were identified in a whole genome manner by ChIP-seq, and inducible *MEF2*C recruitment in the −98 and −148 kb regions of *KLF4* (Fig. 2*A*, and S7 in File S1) was observed. By statin treatment, a 1.2-fold increased binding of *MEF2C* compared with statin (−) was shown in the kb −148 region, and there was a 2.0 fold increase compared with statin (−) for the kb −98 region (Fig. S7 in File S1). The induction rate of −148 kb was lower than −98 kb, reporter assay using deletion mutants showed that the distal binding site at −148 kb contributes more than the −98 kb binding site for statin dependent *KLF4* induction (Fig. 2*B, C*), and the *MEF2* binding motif in the kb −148 region is essential (Fig. 2*D*). Further, considering the co-localization of the epigenetic enhancer marker H3K27ac (Fig. 2*A*), the kb −148 binding site has a fundamental role as a distal enhancer. Since *MEF2C* is reported to exert its activity by the phosphorylation of Ser387 [29], constitutive binding of *MEF2C* in the kb −148 region might be explained by the presence of an inactive form prior to pitavastatin treatment. Further investigation with antibodies that can distinguish between the phosphorylated and non-phosphorylated forms of *MEF2C*, along with a proteomics approach, will ultimately elucidate the detailed dynamics of pitavastatin-induced *MEF2C* modification and binding in the *KLF4* locus.

Active transcription is known to associate with chromatin conformation dynamics, a process that brings enhancers and promoters into proximity of one another [30]. The presence of the modified histone, H3K27ac, and the observed changes in chromatin structure using 3C indicates *KLF4* is transcriptionally regulated by this process. Comprehensive chromatin interaction analysis by ChIA-PET [31] showed the spatial proximity of the kb −148 enhancer with the *KLF4* promoter (Fig. 3*B*), supporting the notion that direct *MEF2C* binding to the distal enhancer participates in chromatin loop formation. The effects on chromatin conformation by statins were evaluated by TaqMan-3C assay (Fig. 3*C*), and the spatial proximity of the promoter and kb −148 enhancer was increased. This observation in millions of cells was confirmed by 3D-FISH (Fig. 4*B, and C*) in single cells. Since the induction of the loop formation has a relationship with transcription complex recruitment efficiency [15], this conformational change may mechanistically contribute to *KLF4* induction. Indirect binding of *MEF2C* to the kb −98 site might be explained by an unidentified binding factor, such as a transcription factor or histone modifier that is active after statin treatment, because there was no *MEF2C*-binding motif in the region (Fig. 2*A*). However, another possibility may be that the kb −98 region was brought adjacent to the promoter by the increased amount of *MEF2C* bound to the −148 kb enhancer (Fig. 2*A* and S7 in File S1). Elucidation of the detailed mechanism of *MEF2C* binding to the −98 kb region will bring about a deeper understanding of statin-related chromatin conformation dynamics.

In summary, a novel *MEF2C* binding site central to *KLF4* induction was identified in the kb −148 region, and this site directly interacts with the promoter region of *KLF4*, despite the long intervening distance in terms of genome sequence. Chromatin interaction analysis and 3D-FISH showed that drug-responsive chromatin conformation change was one of the mechanisms involved in gene induction induced by statins. Further investigation involving a greater number of loci affected by statin treatment through ChIA-PET analyses in combination with 3D-FISH would help elucidate the role of chromatin dynamics in the response to statins.

## Methods

### Cell culture

Human umbilical vein endothelial cells (HUVECs) (Lonza, Basel, Switzerland) were cultured in EGM2MV medium (Lonza), and cells were cultured at 37°C in 5% CO_2_. HUVECs were used within the first 6 passages. For the experimental studies, HUVECs were cultivated in EGM2MV medium containing pitavastatin at a concentration of 1 µM.

### Compounds

Pitavastatin was supplied by Nissan Chemical industries (Tokyo, Japan).

### Animal model

C57BL/6J ApoE−/− mice were purchased from Jackson Laboratory (Bar Harbor, ME). The animal room was controlled at 23 ± 3°C and a relative humidity of 50 ± 20%. Body weight was measured at the time of drug administration. Eight-week-old ApoE deficient mice (n = 12 for each group) were treated for 12 weeks with vehicle alone or pitavastatin at 3mg/kg/treatment with a normal chow diet (CLEA Japan Inc., Tokyo, Japan). The mice were fed twice a day with vehicle or pitavastatin. The mice were anesthetized with an intraperitoneal injection of pentobarbital sodium (50 mg/kg body wt; Kyoritsu Seiyaku,Tokyo, Japan) and the aortae were isolated. All of the aortae were homogenized with ISOGEN solution and the RNA extracted. Total RNA was extracted with a commercially available RNA extraction kit. To determine the levels of expression of *Klf4, Thbd, Nos3* and *β-actin*, real-time quantitative PCR was performed. Regarding Figure S3 in File S1, determination of the plasma cholesterol levels was performed using a commercially available kit (Cholesterol E-Test Wako; Wako Pure Chemical Industries, Osaka, Japan). For histological analyses, after obtaining blood samples from each apoE-KO mouse, 0.9% NaCl was perfused for 5 min through a 20-G injection needle inserted into the left ventricle at a perfusion pressure of 120 cmH_2_O. The same procedure was repeated for 5 min with periodate-lysine-paraformaldehyde (PLP) fixative solution. The heart and aorta were removed rapidly and fixed in PLP fixative solution. Thereafter, the aortic sinus was dissected and embedded in paraffin, and 3-µm cross sections were prepared for Victoria blue-HE staining. The internal elastic lamina was identified under light microscopy (BX50; Olympus, Tokyo, Japan) after Victoria blue-HE staining, and quantification of the plaque lesions was performed using image analysis (Win ROOF; Mitani Co., Tokyo, Japan). All animal experiments were performed in the KOWA company and they were approved by Animal Ethics Committee of Kowa Company.

### Measurement of the pitavastatin concentration in mouse plasma (LC/MS/MS)

An Agilent 1100 system consisting of a degasser, a binary pump, an ALS thermostat, a column compartment set at 30 °C (Agilent Technologies, Santa Clara, CA, USA), an autosampler HTC-PAL set at 20 °C (CTC Analytics, Zwingen, Switzerland) and a TSQ Quantum triple quadrupole mass spectrometry (Thermo Fisher Scientific, Waltham, MA, USA) were used for the LC/MS/MS analysis. Data acquisition and quantification were performed using LCquan (Ver 1.3, Thermo Fisher Scientific). Each mouse was orally administrated pitavastatin once, and the serum average Cmax concentration of 3 mg/kg pitavastatin was 0.26 µM. A sample was extracted from mouse plasma and added to internal standard solution (IS) [32] using liquid-liquid extraction. Pitavastatin and IS were separated on a C_8_ column with a mobile phase that consisted of acetic acid/methanol (4∶6, v/v). Pitavastatin and IS were detected by LC/MS/MS with positive electrospray ionization (ESI). The mass spectrometer was operated in multiple reaction monitoring (MRM) mode using the transition m/z 422–290 for pitavastatin and m/z 406–318.1 for IS, respectively. The standard curve was 6 points (2∼200 ng/mL) and all samples were included in a standard range.

### Gene knock-down by siRNA

Life Technologies negative control siRNA were transfected with lipofectamine 2000 RNAiMAX (Life Technologies/Thermo Fisher Scientific, South San Francisco, CA, USA) according to the manufacturer's protocol. Growth medium was replaced 4 hr after transfection. All of the validated siRNAs were purchased from Life Technologies/Thermo Fisher Scientific.

### Quantitative real-time PCR

RNA was isolated from cells using Isogen reagent (Wako, Osaka, Japan) according to the manufacturer's protocol. Real-time quantitative PCR was performed on a CFX96 detection system (Bio-Rad, Hercules, CA, USA) using TaqMan and SYBR green. The sequences of the applied primers for Human *KLF4, THBD*, *NOS3* and cyclophilin are shown in Table S2B in File S1. The TaqMan probes and primers for Mouse *Klf4, Thbd* and *Nos3* were purchased from Life Technologies/Thermo Fisher Scientific.

### Western blotting

Protein samples were separated by 10% SDS-PAGE and transferred electrophoretically onto nitrocellulose membranes (Hybond-C; GE Healthcare UK Ltd., Buckinghamshire, England). Membranes were blocked with 5% (wt/vol) non-fat milk in PBS containing 0.1% Tween for 1 hour, incubated with antibodies for 1 hour and detected by chemiluminescence using West Dura extended duration substrate (Thermo Fisher Scientific, Waltham, MA, USA) according to the manufacturer's protocol.

### Antibodies

Antibodies recognizing β-actin and α-tubulin (Sigma, St. Louis, MO, USA) as well as MEF2D and nucleoporin p-62 (BD, Franklin Lakes, NJ, USA) were purchased products. Monoclonal antibodies for human *MEF2A, MEF2C, KLF2* and *KLF4* were established with a baculovirus expression system, as described previously [33]. Briefly, mouse IgG mouse monoclonal antibodies against amino acids (aa) 318–404 of human *MEF2*A (IgG-Y0841), (aa) 161–248 of human *MEF2C* (IgG-Y1740), (aa) 2–34 of human *KLF2* (IgG-N2212) and (aa) 26–90 of human *KLF4* (IgG-Y6929) were generated. The recognition of *MEF2A* by Y0841, *MEF2C* by Y1740, *KLF2* by N2212 or *KLF4* by Y6929 was confirmed by immunoblotting using HUVEC proteins. The specificity of these antibodies was confirmed by the reduction of the protein levels using siRNA (Fig. S4 and S5 in File S1). Mouse monoclonal antibody (clone Pd75C9) directed against phosphorylated RNA polymerase II was generated by immunizing mice using a synthetic peptide [SY_1_S_2_P_3_T_4_ (phospho-S)_5_P_6_ (phospho-S)Y_7_SPTSPSYSPC] (Sigma-Genosys) coupled to keyhole limpet hemocyanin, and the specificity was evaluated by ELISA and immunoblotting, as described in Fig. S8*A* and *B* in File S1. [14]

### Comprehensive gene expression analysis

HUVECs were incubated with 1 µM of pitavastatin or DMSO for 4 hours. In this report, we identified genes consistently induced by 4 hour pitavastatin treatment because pitavastatin increased *KLF4* starting at 2 hours and reached a plateau in 6 hours (Fig. S2*A* in File S1). 4 hours of pitavastatin treatment suggested an early phase statin reaction elicited by one of the clinically used statins. The effect on *KLF4* induction by pitavastatin was achieved starting at 0.1 µM and the maximal effect was observed at 1 µM (Fig. S11 in File S1). Therefore, we adopted this concentration in the experiments. We performed the same experiments five times independently for pitavastatin-treatment experiments to eliminate batch-specific differences in drug responsiveness and two times independently for the knockdown experiments. Microarray analyses with siRNA against Control and *KLF4* under 1 µM pitavastatin treatment were performed twice independently. Total cellular RNA was isolated using an RNeasy Micro kit (Qiagen). Preparation of the cRNA and hybridization of the probe arrays were performed according to the manufacturer's instructions (Affymetrix, Santa Clara, CA). Affymetrix GeneChip Human Genome U133 plus 2.0 arrays containing 54,675 probe sets were applied. The expression values for each mRNA were obtained by the Robust Multi-array Analysis (RMA) method [34], [35]. Then the probe sets were classified on the basis of the expression (20.0–100.0) percentile. The probe sets that were expressed at lower than the 20th percentile in all the fourteen arrays were eliminated from the analyses (49,463 probe sets). To analyze expression data based on the gene-level, intensity signal values were summarized using Entrez Gene ID and then averaged (30,344 probe sets). After excluding probe sets which did not have gene symbols, the remaining 20,756 genes were used for further analyses. To identify the genes receiving treatment which had significant changes in expression compared to control treatment, the following selection procedures were performed. Gene expression under pitavastatin treatment was compared with DMSO and gene expression with siRNA against *KLF4* was compared with siControl, then the genes which had a fold change> = 2.0 or < = 0.5 in either comparison were selected (384 genes) (Figure S1 in File S1). Hierarchical clustering analysis was performed using the average linkage and Pearson correlation as a measure of similarity for the selected genes (Fig. 1). All analysis was performed with GeneSpring GX 12.5 (Agilent Technologies, Santa Clara, CA). Annotation of the probe numbers and targeted sequences are shown on the Affymetrix web page.

### Chromatin Immunoprecipitation (ChIP)

The detailed experimental procedure for chromatin immunoprecipitation followed by deep sequencing is described elsewhere [15], [36],[37]. Briefly, 3×10^6^ HUVEC cells were plated on a 15 cm culture plate and cultivated for 3 days. They were treated with pitavastatin at a concentration of 1 µM for 4 hours and cells were crosslinked for 10 min using 1% paraformaldehyde. After neutralization using 0.2 M Glycine solution, cells were recollected and resuspended in SDS lysis buffer (10 mM Tris-HCl (pH 8.0), 150 mM NaCl, 1% SDS, 1 mM EDTA). Cell suspensions, including the DNA-protein complexes, were subjected to fragmentation using a Sonifier-250 (Branson) for 10 min. MEF2C-bound chromatin was isolated from whole cell lysate using an established mouse monoclonal antibody to *MEF2C* in combination with magnetic beads (Life Technologies/Thermo Fisher Scientific). The prepared DNA was quantified using Qubit fluorometer (Life Technologies/Thermo Fisher Scientific) and more than 10 ng of DNA were processed for ChIP-seq. The remaining DNA was used for ChIP-quantitative PCR (qPCR). Primer pairs for ChIP-PCR are shown in Table S2C in File S1.

### ChIP sequencing

All of the protocols for Illumina/Solexa sequencing preparation, sequencing and quality control were provided by Illumina (http://www.illumina.com/pages.ilmn?ID=203) [38].

### Plasmids, transient transfections and luciferase assays

KLF4 promoter fragments were amplified by PCR using genomic DNA from HEK293 as the template. For construction of the KLF4-luc plasmid, the human *KLF4* promoter was cloned into a pGL3-basic vector (Promega, Madison, WI, USA). Primer pairs for construction of *KLF4* promoters are shown in Table S2D in File S1. HUVECs were transiently transfected with plasmid DNA using the Fugene HD reagent (Roche, Mannheim, Germany) and luciferase activity was measured with a dual-luciferase assay kit (Promega) according to the manufacturer's instructions.

### Chromatin interaction analysis with paired-end tag sequencing (ChIA-PET)

Briefly, HUVECs were cultivated and serum starved for 16 hrs. The prepared cells in culture plates were crosslinked using ethylene glycol bis (succinimidylsuccinate) (EGS) (Thermo, USA) at a concentration of 10 mM in 50% glacial acetic acid for 45 min, followed by crosslinking with 1% of paraformaldehyde (TAAB, UK), then neutralized by 2.5 M glycine for 5 min. The harvested cell suspension was sonicated with a Branson sonifier for 4 min 40 sec. Chromatin immunoprecipitation was performed using an antibody for Pol II (Pd75C9) using magnetic beads (Life Technologies/Thermo Fisher Scientific). The detailed library construction procedures and the sequences of the half-linkers were previously reported [31]. In brief, ChIP-DNA fragments immobilized on magnetic beads were end-blunted, then ligated with biotinylated half-linkers. After phosphate group addition to the 5′-ends, the chromatin complex was eluted from the beads, followed by circularization. The circularized products were reverse cross-linked and purified, then digested with Mme I restriction enzyme, which recognizes the half-linker embedded motif. The generated PET products, which contain the full linker in the center and are surrounded 20 base-specific nucleotides at both ends, were immobilized on M-280 Streptavidin Dynabeads (Life Technologies/Thermo Fisher Scientific). Adaptors A and B [31] were ligated to the immobilized PET, and the PET products were amplified as well as quality-controlled using PCR with the Solexa-454 primer [31], then processed by di-tag sequencing using GAII (Illumina). The obtained sequence tags were processed as described previously [16].

### Chromosome Conformation Capture (3C) Assay

The assay was performed utilizing TaqMan 3C Chromosome Conformation Kits (Hind III; cat. #4466152) (Life Technologies) according to the manufacturer's instructions with certain modifications. Briefly, HUVECs were cross-linked with 1% paraformaldehyde for 10 min. The reaction was stopped by the addition of 125 mM glycine for 5 min. Nuclei were re-suspended with restriction enzyme buffer. Samples were treated with 400 U of Hind III at 37°C for 16 hours. After enzymatic digestion, the samples were diluted with ligation buffer and treated with DNA ligase at 16°C for 1 hour. Samples were finally reverse cross-linked and purified. Two human bacterial artificial chromosome (BAC) clones (RP11-150J11, RP11-359L14) were used to normalize the levels of 3C products which may have been PCR-amplified with different primer efficiencies, as mentioned previously [39].

### Two-color 3D fluorescence *in situ* hybridization (3D-FISH) and probe detection

Two BAC clones covering the target regions (the *KLF4* gene and the *MEF2C* binding enhancer) were selected based on a genome database (The UCSC Genome Browser on Human Feb. 2009 (GRCh37/hg19) Assembly). The BAC clones were purchased from Advanced GenoTechs Co. (Tsukuba, Japan) and used as probes. Probe K includes the *KLF4* gene (RP11-80F13) while probe M includes the *MEF2C* binding region (RP11-359L14) (Figs. 3*A* and S10*A* and *L* in File S1). Both of the Probes K and M were labeled using a nick translation kit (Roche) with Dig-11-dUTP and DNP-11-dUTP, respectively, according to the manufacturer's protocol, to measure gene-to-gene distances on each homologous chromosome 9. Approximately 0.125 µg of labeled DNA from each probe was used for each hybridization. 3D-FISH and probe detection were performed according to the protocols described elsewhere with slight modifications [40], [41]. The probe labeled DNAs of the two target regions and Cot-1 DNA (which is used to block repeating elements in order to prevent non-specific hybridization) were mixed and subjected to ethanol precipitation, and then resuspended in hybridization solution (50% formamide and 10% dextran sulfate in 2×Saline Sodium Citrate Buffer (SSC)). The probes were pre-denatured at 80.5°C for 6 min and placed on ice for 1 min. Denatured probes were applied to the coverslips on which cells were fixed, covered with smaller coverslips (18×18 mm), and sealed. The coverslip specimens were denatured at 74°C for 4 min, and hybridization was performed in a moist chamber at 37°C for 3 days. After hybridization, the specimens were washed twice in 2× SSC at room temperature for 5 min, three times in 0.1× SSC at 60°C for 5 min each and blocked with 5% BSA in 4xSSC with 0.2% Tween-20. Immunofluorescence detection was then performed throughout the layers with antibodies diluted to 1∶200 (1st layer: mouse-anti-Dig and rabbit-anti-DNP, 2nd layer: sheep-anti-mouse-Cy3 and goat-anti-rabbit-Alexa488). Nuclear DNA was counterstained with TOPRO-3 (Molecular Probes) and the slides were mounted in Vectashield Antifade (Vector).

### Confocal microscopic Imaging

Nuclei were scanned with a three-channel laser-scanning confocal microscope (LSM510META; Carl Zeiss Co., Ltd.) equipped with a Plan-Apochromat 63×/1.4 Oil DIC objective lens. For each optical section, images were collected sequentially for three fluorochromes (Alexa488, Cy3, and TOPRO-3) using multi-argon (458/477/488/514 nm) and helium-neon (543/633 nm) lasers. To improve the signal-to-noise ratio, each sectional image obtained was an average of eight successive scans. The z-step between sections was 0.2 µm. Stacks of 12-bit gray scale two-dimensional images were obtained with 512×512 pixels in each channel. Confocal image stacks were processed with microscope operating software (LSM5; Carl ZeissMicroImaging GmbH) and saved as LSM files. More than 50 nuclear images were captured from each of the cellular materials. Nuclei from cultured cells with singlet-singlet signals were adopted for calculation as in the G1 phase of the cell cycle, but with doublet-doublet or singlet-doublet signals for each probe, which were suspected of being in the S or G2 phase, were not selected for capture. The representative 3D-images shown in Figure 4*B* were processed and reconstructed with Imaris 7.5 (Bitplane Scientific Software).

### Quantitative 3D evaluation

The image stacks were 3D-reconstructed using the program AVS/Express version 7.2 and the reasonable thresholds for each channel were determined by a 3D coordination of the whole volume of the gene signals. The 3D coordinates of two target regions in each cell were specified and the shortest distance between the gravity center of each gene was calculated using 3D image processing and analysis software CTMS (Chromosome Territory Measurement Software) (Cybernet Co. Ltd.).

### Statistical analysis

Values are reported as the mean ± standard deviation (SD). Student's t-test, Dunnett's test, or Wilcoxon rank-sum test was used for comparisons between two groups. Differences were considered statistically significant when the *P*-value was less than 0.05.

All of the microarray data files, ChIP-seq data files and ChIA-PET data file are deposited. The GEO and accession numbers are: GSE32547, GSE32644, GSE32693, GSE41553.

## Supporting Information

File S1 **Supporting Information. Figure S1. Flow chart for microarray analysis.** Affymetrix GeneChip Human Genome U133 plus 2.0 arrays were applied for all analysis. The analysis was performed with GeneSpring GX 12.5 following the layout on this flow chart. The details are shown in the *Methods* section. Insignificant or unannotated probe data was eliminated at each step. The number on the right side shows the number of the remaining probe sets or genes at each step. The 384 selected genes were used for further analyses in Figure 1 and Table S1 in File S1. **Figure S2. Gene regulation by pitavastatin in HUVECs and the aortae of Apo-E-deficient mice.** (A) HUVECs were treated with 1 µM pitavastatin for the indicated time. (B) ApoE deficient mice were orally administered pitavastatin twice daily at 3 mg/kg/treatment for 12 weeks before sacrifice. Total RNA was isolated and determined by real-time quantitative PCR, as described in *Methods*. The sequences of applied primers are shown in Table S2B in File S1. Vertical lines indicate the S.D. (n = 3 in A, and n = 12 in B), * *P*<0.01, ** *P*<0.001, compared with the control sample, Dunnett's test in A and Student's t test in B. **Figure S3. Histological examination of atherosclerotic regions in the aortic sinus.** Eight-week-old ApoE deficient mice (n = 12 for each group) were treated twice daily for 12 weeks with vehicle alone (-) or pitavastatin at 3 mg/kg/treatment (+). The heart and aorta were removed rapidly and fixed and embedded in paraffin for Victoria blue-HE staining as described in *Methods*. Victoria blue-hematoxylin-eosin staining (A) revealed the atherosclerotic lesions (arrow). (B) shows the total plasma cholesterol and triglyceride levels. Note that pitavastatin reduced the plaque area without changing the plasma cholesterol and triglyceride levels. The vertical lines indicate the SEM (*n* = 12), * *P*<0.001 compared with the control sample, Student's t test. n.s. indicates not significant. **Figure S4. Identification of the** ***MEF2A, MEF2C, KLF2*** **and** ***KLF4*** **antibodies.** HUVECs were incubated with 1 µM pitavastatin for 4 hours. Before pitavastatin treatment, as described in the *Methods* section, cells were transfected with siRNA to *KLF2* (A), *KLF4* (B), *MEF2A* (C) and *MEF2C* (D). (A, C, and D) The whole cell fraction was prepared for the Western blotting experiment to detect *KLF2*, *MEF2A* and *MEF2C*. Beta-actin was used as the internal control. (B) HUVECs were incubated with 1 µM pitavastatin for 24 hours. The nuclear extract fraction was prepared for further Western blotting to detect *KLF4*. Nucleoporin p62 was used as an internal control. NS; Non silencing for negative control. Note that loss of band detection was observable only by treatment specific siRNA, which demonstrates the specificity of new antibodies. N2212, Y6929, Y0841, Y1740 are clone numbers for each antibody. **Figure S5. Triple knock down of** ***MEF2A, C*** **and** ***D*** **reduces** ***KLF2*** **and** ***KLF4*** **.** HUVECs were incubated with 1 µM pitavastatin for 4 hours. Before the treatment, as described in the *Methods* section, cells were transfected with siRNA to *MEF2A, -2C* and *-2D*. A whole cell fraction was prepared for a further Western blotting experiment to detect *KLF2*, and a nuclear extract fraction was prepared for *KLF4*. Alpha Tubulin and Nucleoporin p62 were used as the internal controls for the whole cell lysate and nuclear extract fractions, respectively. NS; Non silencing for negative control. **Figure S6. Localization of the** ***MEF2C*** **binding sites in HUVECs.** HUVECs were incubated with DMSO [statin(-)]or 1 µM pitavastatin for 4 hours. (A) Chromatin immunoprecipitation was performed using an *MEF2C* antibody, followed by deep sequencing. 4,878 *MEF2C* binding sites were detected in the control [pitavastatin (−)]. 42% of the *MEF2C* binding sites were located between the TSS and 3′UTR of the genes, while the remaining of 58% were found in intergenic regions. (B) Co-localization of *MEF2C* binding sites and H3K27ac in control [pitavastatin (−)] HUVECs. 25,477 binding sites were detected using anti-H3K27ac antibody ChIP-seq analysis in the control [pitavastatin (−)] HUVECs. Among them, 798 binding sites displayed co-localization of H3K27ac and *MEF2C*. (C) Distribution of the *MEF2C* binding sites in the pitavastatin-treated HUVECs. 13,030 MEF2C binding sites were detected in the treated HUVECs; 40% of them were located between the TSS and 3′UTR of the genes, while 60% were located in intergenic regions. **Figure S7. ChIP-qPCR with an anti-** ***MEF2C*** **antibody against the** ***KLF4*** **gene.** *MEF2C* Binding sites detected in the *KLF4* upstream region (Figure 2A) were quantitatively evaluated by ChIP-qPCR. The Kb –147 *KLF4* region was used as the negative control. The sequences of the primers are shown in Table S2C in File S1. Vertical lines indicate the S.D. (*n* = 3), *P<0.01, compared with the kb −147 *KLF4*, statin (−), ‡*P*<0.01 compared with the kb −98 or kb-148 *KLF4*, statin (−), Student's t test. **Figure S8. The specificity of the Pol II antibody.** A newly-developed monoclonal antibody (Pd75C9) was used to perform ELISA with RNA polymerase II C-terminal domain peptides containing different phosphorylation patterns. (A) The list of peptides used for ELISA. Phosphorylated amino acids are indicated in red. C-terminal repeat of RNA Polymerase II is comprises from 25 to 52 tandem copies of the consensus repeat heptad Y_1_S_2_P_3_T_4_S_5_P_6_S_7_ (Also shown in the right panel of figure A). And antibodies for a variety of phosphorylated CTD were raised using 19 AA peptides including phosphorylated Serine as is depicted. (B) ELISA. Microtiter plates coated with the peptides (the sequence shown in A) were incubated with 3-fold dilutions of the monoclonal antibody (starting from a 1∶27 dilution of a hybridoma culture supernatant). After incubation with a peroxidase-conjugated secondary antibody and washing, the colorimetric signal of tetramethylbenzidine was detected by measuring the absorbance at 405 nm (Abs) using a plate reader. Although Pd75C9 was generated using the Pd peptide to immunize mice, this clone reacted with all of the phospho-serine containing peptides, with preference for those containing phospho-S5 and phosphor-S2, but reacted only slightly with the unphosphorylated peptides. **Figure S9. Sequence of the PCR product generated in the quantitative 3C assay.** To validate the quantitative 3C assay, PCR products were directly sequenced and identified. The chromatogram shows the sequence of the target analysis (the fragments including *KLF4* and 148 kb upstream from the TSS of *KLF4*) under pitavastatin treatment. As expected, the sequence of the TSS and that of the kb −148 enhancer were directly connected by a Hind III recognition sequence, and the measurement of the amount of this PCR product stands for the frequency of chromatin interaction. **Figure S10. The sensitivity and specificity of the probes.** To validate the probes, BAC clone DNA (RP11-80F13 and RP11-359L14) was tested for correct chromosomal location at 9q31.2 and the absence of other signals by 2D-FISH, respectively. The following datafrom (A) to (K) is the detailed information on probe K and (L) to (Q) is the detailed information of probe M. (A) Probe design for the 2D-FISH analysis of the target region on human Chr.9q31.2. The numbers in the middle indicate the location on Chr.9 using the hg19 build program. Probe K includes the *KLF4* gene. Three-color 2D-FISH was carried out by a combination of a labeled Probe K and human Chr.9 arm-specific painting probes (courtesy of Prof. Dr. T. Cremer, LMU, Munich). (B) The p arm of Chr.9 is represented in purple. (C) The q arm of Chr.9 represented in green. (D) Probe K is represented in red. (E) Nuclear DNA was counterstained with DAPI (4′, 6-diamidino-2-phenylindole) and is shown in blue. The merged image with all of the colors is shown in (F). Probe K in the interphase is shown in Figures G to K. All of the combinations of the labeled Probe K and human Chr.9 p and q arm-specific painting probes were the same as in B-F. The white arrows indicate the representative signals of Probe K in the interphase (K). (L) Probe design for the 2D-FISH analysis of the target region on human Chr.9q31.2. The numbers in the middle indicate the location on Chr.9 using the hg19 build program. Probe M includes the *MEF2C* binding region, which is located 148 kb upstream from the TSS of *KLF4*. (M) The q arm of Chr.9 is represented in red. (N) The p arm of Chr.9 is represented in purple. (O) Probe M is represented in green. (P) Nuclear DNA was counterstained with DAPI (4′, 6-diamidino-2-phenylindole) and is represented in blue. The merged image with all of the colors is shown in (Q). The white arrows indicate the representative signals of Probe M in the metaphase. The signals in the interphase are shown in the other nuclei. It was confirmed that the signal of the BAC clone was clearly detected on the Chr.9q region and absent from the other chromosomes. **Figure S11. Statin effect on** ***KLF4*** **expression.** HUVECs were treated with pitavastatin at a concentration ranging from 0.01 to 10 µM for 12 hours. Total RNA was isolated and determined by Real-time quantitative PCR, as described in the *Methods* section. The vertical lines indicate the S.D. (n = 3), * *P*<0.001, compared with the sample of 0 µM, Dunnett's test. **Table S1. Table S2**.
(PDF)

## References

[1] Endo A, Kuroda M, Tsujita Y (1976) ML-236A, ML-236B, and ML-236C, new inhibitors of cholesterogenesis produced by Penicillium citrinium. J Antibiot (Tokyo) 29: 1346–1348. 1010803 10.7164/antibiotics.29.1346

[2] Goldstein JL, Brown MS (2009) The LDL receptor. Arterioscler Thromb Vasc Biol 29: 431–438. 19299327 10.1161/ATVBAHA.108.179564 PMC2740366

[3] Teramoto T, Shimano H, Yokote K, Urashima M (2009) Effects of pitavastatin (LIVALO Tablet) on high density lipoprotein cholesterol (HDL-C) in hypercholesterolemia. J Atheroscler Thromb 16: 654–661. 19907105 10.5551/jat.1719

[4] Schonbeck U, Libby P (2004) Inflammation, immunity, and HMG-CoA reductase inhibitors: statins as antiinflammatory agents? Circulation 109: II18–26. 15173059 10.1161/01.CIR.0000129505.34151.23

[5] Sen-Banerjee S, Mir S, Lin Z, Hamik A, Atkins GB, et al (2005) Kruppel-like factor 2 as a novel mediator of statin effects in endothelial cells. Circulation 112: 720–726. 16043642 10.1161/CIRCULATIONAHA.104.525774

[6] Parmar KM, Nambudiri V, Dai G, Larman HB, Gimbrone MA Jr, et al (2005) Statins exert endothelial atheroprotective effects via the KLF2 transcription factor. J Biol Chem 280: 26714–26719. 15878865 10.1074/jbc.C500144200

[7] Das H, Kumar A, Lin Z, Patino WD, Hwang PM, et al (2006) Kruppel-like factor 2 (KLF2) regulates proinflammatory activation of monocytes. Proc Natl Acad Sci U S A 103: 6653–6658. 16617118 10.1073/pnas.0508235103 PMC1458936

[8] SenBanerjee S, Lin Z, Atkins GB, Greif DM, Rao RM, et al (2004) KLF2 Is a novel transcriptional regulator of endothelial proinflammatory activation. J Exp Med 199: 1305–1315. 15136591 10.1084/jem.20031132 PMC2211816

[9] Atkins GB, Jain MK (2007) Role of Kruppel-like transcription factors in endothelial biology. Circ Res 100: 1686–1695. 17585076 10.1161/01.RES.0000267856.00713.0a

[10] Dekker RJ, van Soest S, Fontijn RD, Salamanca S, de Groot PG, et al (2002) Prolonged fluid shear stress induces a distinct set of endothelial cell genes, most specifically lung Kruppel-like factor (KLF2). Blood 100: 1689–1698. 12176889 10.1182/blood-2002-01-0046

[11] Parmar KM, Larman HB, Dai G, Zhang Y, Wang ET, et al (2006) Integration of flow-dependent endothelial phenotypes by Kruppel-like factor 2. J Clin Invest 116: 49–58. 16341264 10.1172/JCI24787 PMC1307560

[12] Packard RR, Libby P (2008) Inflammation in atherosclerosis: from vascular biology to biomarker discovery and risk prediction. Clin Chem 54: 24–38. 18160725 10.1373/clinchem.2007.097360

[13] Libby P (2012) Inflammation in atherosclerosis. Arterioscler Thromb Vasc Biol 32: 2045–2051. 22895665 10.1161/ATVBAHA.108.179705 PMC3422754

[14] Kimura H, Hayashi-Takanaka Y, Goto Y, Takizawa N, Nozaki N (2008) The organization of histone H3 modifications as revealed by a panel of specific monoclonal antibodies. Cell Struct Funct 33: 61–73. 18227620 10.1247/csf.07035

[15] Kanki Y, Kohro T, Jiang S, Tsutsumi S, Mimura I, et al (2011) Epigenetically coordinated GATA2 binding is necessary for endothelium-specific endomucin expression. EMBO J 30: 2582–2595. 21666600 10.1038/emboj.2011.173 PMC3155306

[16] Li G, Fullwood MJ, Xu H, Mulawadi FH, Velkov S, et al (2010) ChIA-PET tool for comprehensive chromatin interaction analysis with paired-end tag sequencing. Genome biology 11: R22. 20181287 10.1186/gb-2010-11-2-r22 PMC2872882

[17] Li G, Ruan X, Auerbach RK, Sandhu KS, Zheng M, et al (2012) Extensive promoter-centered chromatin interactions provide a topological basis for transcription regulation. Cell 148: 84–98. 22265404 10.1016/j.cell.2011.12.014 PMC3339270

[18] McConnell BB, Yang VW (2010) Mammalian Kruppel-like factors in health and diseases. Physiological reviews 90: 1337–1381. 20959618 10.1152/physrev.00058.2009 PMC2975554

[19] Hamik A, Lin Z, Kumar A, Balcells M, Sinha S, et al (2007) Kruppel-like factor 4 regulates endothelial inflammation. J Biol Chem 282: 13769–13779. 17339326 10.1074/jbc.M700078200

[20] Sabatine MS, Ploughman L, Simonsen KL, Iakoubova OA, Kirchgessner TG, et al (2008) Association between ADAMTS1 matrix metalloproteinase gene variation, coronary heart disease, and benefit of statin therapy. Arterioscler Thromb Vasc Biol 28: 562–567. 18174457 10.1161/ATVBAHA.107.156653

[21] Villarreal G Jr, Zhang Y, Larman HB, Gracia-Sancho J, Koo A, et al (2010) Defining the regulation of KLF4 expression and its downstream transcriptional targets in vascular endothelial cells. Biochemical and biophysical research communications 391: 984–989. 19968965 10.1016/j.bbrc.2009.12.002 PMC4165389

[22] Jougasaki M, Ichiki T, Takenoshita Y, Setoguchi M (2010) Statins suppress interleukin-6-induced monocyte chemo-attractant protein-1 by inhibiting Janus kinase/signal transducers and activators of transcription pathways in human vascular endothelial cells. British journal of pharmacology 159: 1294–1303. 20136831 10.1111/j.1476-5381.2009.00612.x PMC2848933

[23] Wang X, Merritt AJ, Seyfried J, Guo C, Papadakis ES, et al (2005) Targeted deletion of mek5 causes early embryonic death and defects in the extracellular signal-regulated kinase 5/myocyte enhancer factor 2 cell survival pathway. Mol Cell Biol 25: 336–345. 15601854 10.1128/MCB.25.1.336-345.2005 PMC538774

[24] Hayashi M, Kim SW, Imanaka-Yoshida K, Yoshida T, Abel ED, et al (2004) Targeted deletion of BMK1/ERK5 in adult mice perturbs vascular integrity and leads to endothelial failure. J Clin Invest 113: 1138–1148. 15085193 10.1172/JCI19890 PMC385403

[25] Lin Q, Lu J, Yanagisawa H, Webb R, Lyons GE, et al (1998) Requirement of the MADS-box transcription factor MEF2C for vascular development. Development 125: 4565–4574. 9778514 10.1242/dev.125.22.4565

[26] Kato Y, Zhao M, Morikawa A, Sugiyama T, Chakravortty D, et al (2000) Big mitogen-activated kinase regulates multiple members of the MEF2 protein family. J Biol Chem 275: 18534–18540. 10849446 10.1074/jbc.M001573200

[27] Arnold MA, Kim Y, Czubryt MP, Phan D, McAnally J, et al (2007) MEF2C transcription factor controls chondrocyte hypertrophy and bone development. Dev Cell 12: 377–389. 17336904 10.1016/j.devcel.2007.02.004

[28] Wang L, Fan C, Topol SE, Topol EJ, Wang Q (2003) Mutation of MEF2A in an inherited disorder with features of coronary artery disease. Science 302: 1578–1581. 14645853 10.1126/science.1088477 PMC1618876

[29] Han J, Molkentin JD (2000) Regulation of MEF2 by p38 MAPK and its implication in cardiomyocyte biology. Trends Cardiovasc Med 10: 19–22. 11150724 10.1016/s1050-1738(00)00039-6

[30] Dekker J, Rippe K, Dekker M, Kleckner N (2002) Capturing chromosome conformation. Science 295: 1306–1311. 11847345 10.1126/science.1067799

[31] Fullwood MJ, Liu MH, Pan YF, Liu J, Xu H, et al (2009) An oestrogen-receptor-alpha-bound human chromatin interactome. Nature 462: 58–64. 19890323 10.1038/nature08497 PMC2774924

[32] Kojima J, Fujino H, Yosimura M, Morikawa H, Kimata H (1999) Simultaneous determination of NK-104 and its lactone in biological samples by column-switching high-performance liquid chromatography with ultraviolet detection. J Chromatogr B Biomed Sci Appl 724: 173–180. 10202970 10.1016/s0378-4347(98)00523-4

[33] Jiang S, Tanaka T, Iwanari H, Hotta H, Yamashita H, et al (2003) Expression and localization of P1 promoter-driven hepatocyte nuclear factor-4alpha (HNF4alpha) isoforms in human and rats. Nucl Recept 1: 5. 12952540 10.1186/1478-1336-1-5 PMC194242

[34] Irizarry RA, Hobbs B, Collin F, Beazer-Barclay YD, Antonellis KJ, et al (2003) Exploration, normalization, and summaries of high density oligonucleotide array probe level data. Biostatistics 4: 249–264. 12925520 10.1093/biostatistics/4.2.249

[35] Shakya K, Ruskin HJ, Kerr G, Crane M, Becker J (2010) Comparison of microarray preprocessing methods. Adv Exp Med Biol 680: 139–147. 20865495 10.1007/978-1-4419-5913-3_16

[36] Wada Y, Ohta Y, Xu M, Tsutsumi S, Minami T, et al (2009) A wave of nascent transcription on activated human genes. Proc Natl Acad Sci U S A 106: 18357–18361. 19826084 10.1073/pnas.0902573106 PMC2761237

[37] Tozawa H, Kanki Y, Suehiro J, Tsutsumi S, Kohro T, et al (2011) Genome-wide approaches reveal functional interleukin-4-inducible STAT6 binding to the vascular cell adhesion molecule 1 promoter. Mol Cell Biol 31: 2196–2209. 21464207 10.1128/MCB.01430-10 PMC3133239

[38] Seila AC, Calabrese JM, Levine SS, Yeo GW, Rahl PB, et al (2008) Divergent transcription from active promoters. Science 322: 1849–1851. 19056940 10.1126/science.1162253 PMC2692996

[39] Hagege H, Klous P, Braem C, Splinter E, Dekker J, et al (2007) Quantitative analysis of chromosome conformation capture assays (3C-qPCR). Nature protocols 2: 1722–1733. 17641637 10.1038/nprot.2007.243

[40] Kawamura R, Tanabe H, Wada T, Saitoh S, Fukushima Y, et al (2012) Visualization of the spatial positioning of the SNRPN, UBE3A, and GABRB3 genes in the normal human nucleus by three-color 3D fluorescence in situ hybridization. Chromosome research: an international journal on the molecular, supramolecular and evolutionary aspects of chromosome biology 10.1007/s10577-012-9300-5 PMC3481056 22801776

[41] Tanabe H, Muller S, Neusser M, von Hase J, Calcagno E, et al (2002) Evolutionary conservation of chromosome territory arrangements in cell nuclei from higher primates. Proc Natl Acad Sci U S A 99: 4424–4429. 11930003 10.1073/pnas.072618599 PMC123664

