# Correction: Direct Evidence for Pitavastatin Induced Chromatin Structure Change in the *KLF4* Gene in Endothelial Cells

**DOI:** 10.1371/journal.pone.0099749

**Published:** 2014-06-02

**Authors:** The PLOS ONE Staff

Some of the Supporting information was erroneously omitted from the publication. Please view the corrected Supporting Information in its entirety here.

## Supporting Information

File S1 **Supporting Information. Figure S1. Flow chart for microarray analysis.** Affymetrix GeneChip Human Genome U133 plus 2.0 arrays were applied for all analysis. The analysis was performed with GeneSpring GX 12.5 following the layout on this flow chart. The details are shown in the *Methods* section. Insignificant or unannotated probe data was eliminated at each step. The number on the right side shows the number of the remaining probe sets or genes at each step. The 384 selected genes were used for further analyses in Figure 1 and Table S1 in File S1. **Figure S2. Gene regulation by pitavastatin in HUVECs and the aortae of Apo-E-deficient mice.** (A) HUVECs were treated with 1 µM pitavastatin for the indicated time. (B) ApoE deficient mice were orally administered pitavastatin twice daily at 3 mg/kg/treatment for 12 weeks before sacrifice. Total RNA was isolated and determined by real-time quantitative PCR, as described in *Methods*. The sequences of applied primers are shown in Table S2B in File S1. Vertical lines indicate the S.D. (n  =  3 in A, and n  =  12 in B), * *P*<0.01, ** *P*<0.001, compared with the control sample, Dunnett's test in A and Student's t test in B. **Figure S3. Histological examination of atherosclerotic regions in the aortic sinus. **Eight-week-old ApoE deficient mice (n  =  12 for each group) were treated twice daily for 12 weeks with vehicle alone (-) or pitavastatin at 3 mg/kg/treatment (+). The heart and aorta were removed rapidly and fixed and embedded in paraffin for Victoria blue-HE staining as described in *Methods*. Victoria blue-hematoxylin-eosin staining (A) revealed the atherosclerotic lesions (arrow). (B) shows the total plasma cholesterol and triglyceride levels. Note that pitavastatin reduced the plaque area without changing the plasma cholesterol and triglyceride levels. The vertical lines indicate the SEM (*n*  =  12), * *P*<0.001 compared with the control sample, Student's t test. n.s. indicates not significant. **Figure S4. Identification of the** ***MEF2A, MEF2C, KLF2*** **and** ***KLF4*** **antibodies. **HUVECs were incubated with 1 µM pitavastatin for 4 hours. Before pitavastatin treatment, as described in the *Methods* section, cells were transfected with siRNA to *KLF2* (A), *KLF4* (B), *MEF2A* (C) and *MEF2C* (D). (A, C, and D) The whole cell fraction was prepared for the Western blotting experiment to detect *KLF2*, *MEF2A* and *MEF2C*. Beta-actin was used as the internal control. (B) HUVECs were incubated with 1 µM pitavastatin for 24 hours. The nuclear extract fraction was prepared for further Western blotting to detect *KLF4*. Nucleoporin p62 was used as an internal control. NS; Non silencing for negative control. Note that loss of band detection was observable only by treatment specific siRNA, which demonstrates the specificity of new antibodies. N2212, Y6929, Y0841, Y1740 are clone numbers for each antibody. **Figure S5. Triple knock down of** ***MEF2A, C*** **and** ***D*** **reduces** ***KLF2*** **and** ***KLF4*** **.** HUVECs were incubated with 1 µM pitavastatin for 4 hours. Before the treatment, as described in the *Methods* section, cells were transfected with siRNA to *MEF2A, -2C* and *-2D*. A whole cell fraction was prepared for a further Western blotting experiment to detect *KLF2*, and a nuclear extract fraction was prepared for *KLF4*. Alpha Tubulin and Nucleoporin p62 were used as the internal controls for the whole cell lysate and nuclear extract fractions, respectively. NS; Non silencing for negative control. **Figure S6. Localization of the** ***MEF2C*** **binding sites in HUVECs. **HUVECs were incubated with DMSO [statin(-)]or 1 µM pitavastatin for 4 hours. (A) Chromatin immunoprecipitation was performed using an *MEF2C* antibody, followed by deep sequencing. 4,878 *MEF2C* binding sites were detected in the control [pitavastatin (−)]. 42% of the *MEF2C* binding sites were located between the TSS and 3′UTR of the genes, while the remaining of 58% were found in intergenic regions. (B) Co-localization of *MEF2C* binding sites and H3K27ac in control [pitavastatin (−)] HUVECs. 25,477 binding sites were detected using anti-H3K27ac antibody ChIP-seq analysis in the control [pitavastatin (−)] HUVECs. Among them, 798 binding sites displayed co-localization of H3K27ac and *MEF2C*. (C) Distribution of the *MEF2C* binding sites in the pitavastatin-treated HUVECs. 13,030 MEF2C binding sites were detected in the treated HUVECs; 40% of them were located between the TSS and 3′UTR of the genes, while 60% were located in intergenic regions. **Figure S7. ChIP-qPCR with an anti-** ***MEF2C*** **antibody against the** ***KLF4*** **gene.** *MEF2C* Binding sites detected in the *KLF4* upstream region (Figure 2A) were quantitatively evaluated by ChIP-qPCR. The Kb –147 *KLF4* region was used as the negative control. The sequences of the primers are shown in Table S2C in File S1. Vertical lines indicate the S.D. (*n*  =  3), *P<0.01, compared with the kb −147 *KLF4*, statin (−), ‡*P*<0.01 compared with the kb −98 or kb-148 *KLF4*, statin (−), Student's t test. **Figure S8. The specificity of the Pol II antibody.** A newly-developed monoclonal antibody (Pd75C9) was used to perform ELISA with RNA polymerase II C-terminal domain peptides containing different phosphorylation patterns. (A) The list of peptides used for ELISA. Phosphorylated amino acids are indicated in red. C-terminal repeat of RNA Polymerase II is comprises from 25 to 52 tandem copies of the consensus repeat heptad Y_1_S_2_P_3_T_4_S_5_P_6_S_7_ (Also shown in the right panel of figure A). And antibodies for a variety of phosphorylated CTD were raised using 19 AA peptides including phosphorylated Serine as is depicted. (B) ELISA. Microtiter plates coated with the peptides (the sequence shown in A) were incubated with 3-fold dilutions of the monoclonal antibody (starting from a 1:27 dilution of a hybridoma culture supernatant). After incubation with a peroxidase-conjugated secondary antibody and washing, the colorimetric signal of tetramethylbenzidine was detected by measuring the absorbance at 405 nm (Abs) using a plate reader. Although Pd75C9 was generated using the Pd peptide to immunize mice, this clone reacted with all of the phospho-serine containing peptides, with preference for those containing phospho-S5 and phosphor-S2, but reacted only slightly with the unphosphorylated peptides. **Figure S9. Sequence of the PCR product generated in the quantitative 3C assay.** To validate the quantitative 3C assay, PCR products were directly sequenced and identified. The chromatogram shows the sequence of the target analysis (the fragments including *KLF4* and 148 kb upstream from the TSS of *KLF4*) under pitavastatin treatment. As expected, the sequence of the TSS and that of the kb −148 enhancer were directly connected by a Hind III recognition sequence, and the measurement of the amount of this PCR product stands for the frequency of chromatin interaction. **Figure S10. The sensitivity and specificity of the probes.** To validate the probes, BAC clone DNA (RP11-80F13 and RP11-359L14) was tested for correct chromosomal location at 9q31.2 and the absence of other signals by 2D-FISH, respectively. The following datafrom (A) to (K) is the detailed information on probe K and (L) to (Q) is the detailed information of probe M. (A) Probe design for the 2D-FISH analysis of the target region on human Chr.9q31.2. The numbers in the middle indicate the location on Chr.9 using the hg19 build program. Probe K includes the *KLF4* gene. Three-color 2D-FISH was carried out by a combination of a labeled Probe K and human Chr.9 arm-specific painting probes (courtesy of Prof. Dr. T. Cremer, LMU, Munich). (B)The p arm of Chr.9 is represented in purple. (C) The q arm of Chr.9 represented in green. (D) Probe K is represented in red. (E) Nuclear DNA was counterstained with DAPI (4′, 6-diamidino-2-phenylindole) and is shown in blue. The merged image with all of the colors is shown in (F). Probe K in the interphase is shown in Figures G to K. All of the combinations of the labeled Probe K and human Chr.9 p and q arm-specific painting probes were the same as in B-F. The white arrows indicate the representative signals of Probe K in the interphase (K). (L) Probe design for the 2D-FISH analysis of the target region on human Chr.9q31.2. The numbers in the middle indicate the location on Chr.9 using the hg19 build program. Probe M includes the *MEF2C* binding region, which is located 148 kb upstream from the TSS of *KLF4*. (M) The q arm of Chr.9 is represented in red. (N) The p arm of Chr.9 is represented in purple. (O) Probe M is represented in green. (P) Nuclear DNA was counterstained with DAPI (4′, 6-diamidino-2-phenylindole) and is represented in blue. The merged image with all of the colors is shown in (Q). The white arrows indicate the representative signals of Probe M in the metaphase. The signals in the interphase are shown in the other nuclei. It was confirmed that the signal of the BAC clone was clearly detected on the Chr.9q region and absent from the other chromosomes. **Figure S11. Statin effect on** ***KLF4*** **expression. **HUVECs were treated with pitavastatin at a concentration ranging from 0.01 to 10 µM for 12 hours. Total RNA was isolated and determined by Real-time quantitative PCR, as described in the *Methods* section. The vertical lines indicate the S.D. (n  =  3), * *P*<0.001, compared with the sample of 0 µM, Dunnett's test. Table S1. Table S2.
(PDF)
