# Supplementary material for: Direct Evidence for Pitavastatin Induced Chromatin Structure Change in the KLF4 Gene in Endothelial Cells
Source: PLoS One. 2014 May 5;9(5):e96005. doi: 10.1371/journal.pone.0096005 (PMC4010393; doi:10.1371/journal.pone.0096005)
Supplement: File S1 — Supporting Information. Figure S1. Flow chart for microarray analysis. Affymetrix GeneChip Human Genome U133 plus 2.0 arrays were applied for all analysis. The analysis was performed with GeneSpring GX 12.5 following the layout on this flow chart. The details are shown in the Methods section. Insignificant or unannotated probe data was eliminated at each step. The number on the right side shows the number of the remaining probe sets or genes at each step. The 384 selected genes were used for further analyses in Figure 1 and Table S1 in File S1. Figure S2. Gene regulation by pitavastatin in HUVECs and the aortae of Apo-E-deficient mice. (A) HUVECs were treated with 1 µM pitavastatin for the indicated time. (B) ApoE deficient mice were orally administered pitavastatin twice daily at 3 mg/kg/treatment for 12 weeks before sacrifice. Total RNA was isolated and determined by real-time quantitative PCR, as described in Methods. The sequences of applied primers are shown in Table S2B in File S1. Vertical lines indicate the S.D. (n = 3 in A, and n = 12 in B), * P<0.01, ** P<0.001, compared with the control sample, Dunnett's test in A and Student's t test in B. Figure S3. Histological examination of atherosclerotic regions in the aortic sinus. Eight-week-old ApoE deficient mice (n = 12 for each group) were treated twice daily for 12 weeks with vehicle alone (-) or pitavastatin at 3 mg/kg/treatment (+). The heart and aorta were removed rapidly and fixed and embedded in paraffin for Victoria blue-HE staining as described in Methods. Victoria blue-hematoxylin-eosin staining (A) revealed the atherosclerotic lesions (arrow). (B) shows the total plasma cholesterol and triglyceride levels. Note that pitavastatin reduced the plaque area without changing the plasma cholesterol and triglyceride levels. The vertical lines indicate the SEM (n = 12), * P<0.001 compared with the control sample, Student's t test. n.s. indicates not significant. Figure S4. Identification of the MEF2A, MEF2C, [file pone.0096005.s001.pdf]

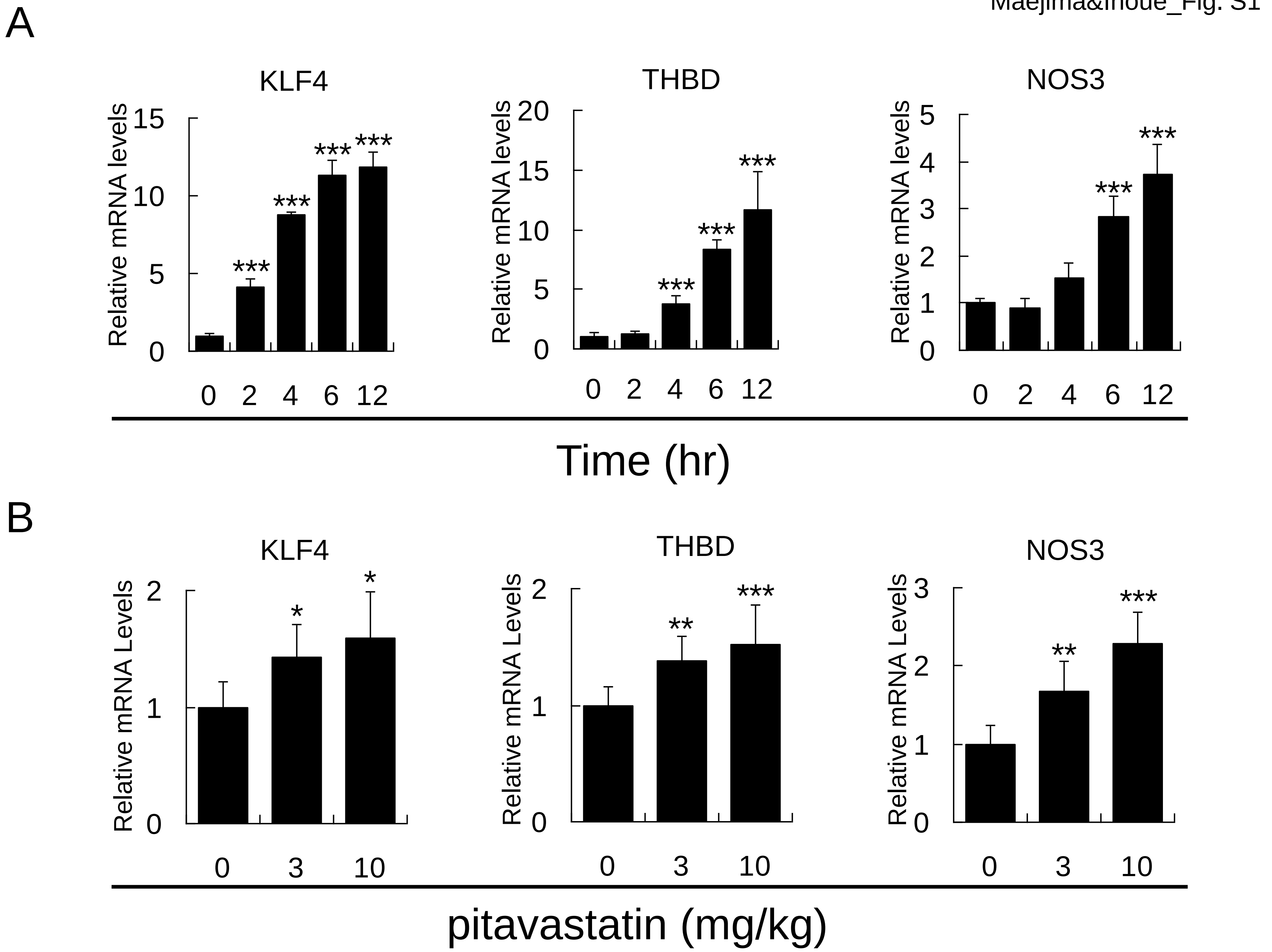
A
KLF4
THBD
NOS3
Relative mRNA levels
0
5
10
15
20
1
2
3
4
***
0 2 4 6 12
Time (hr)
B
KLF4
THBD
NOS3
Relative mRNA Levels
0
1
2
3
*
**
***
0 3 10
pitavastatin (mg/kg)

Maejima&Inoue_Fig. S2

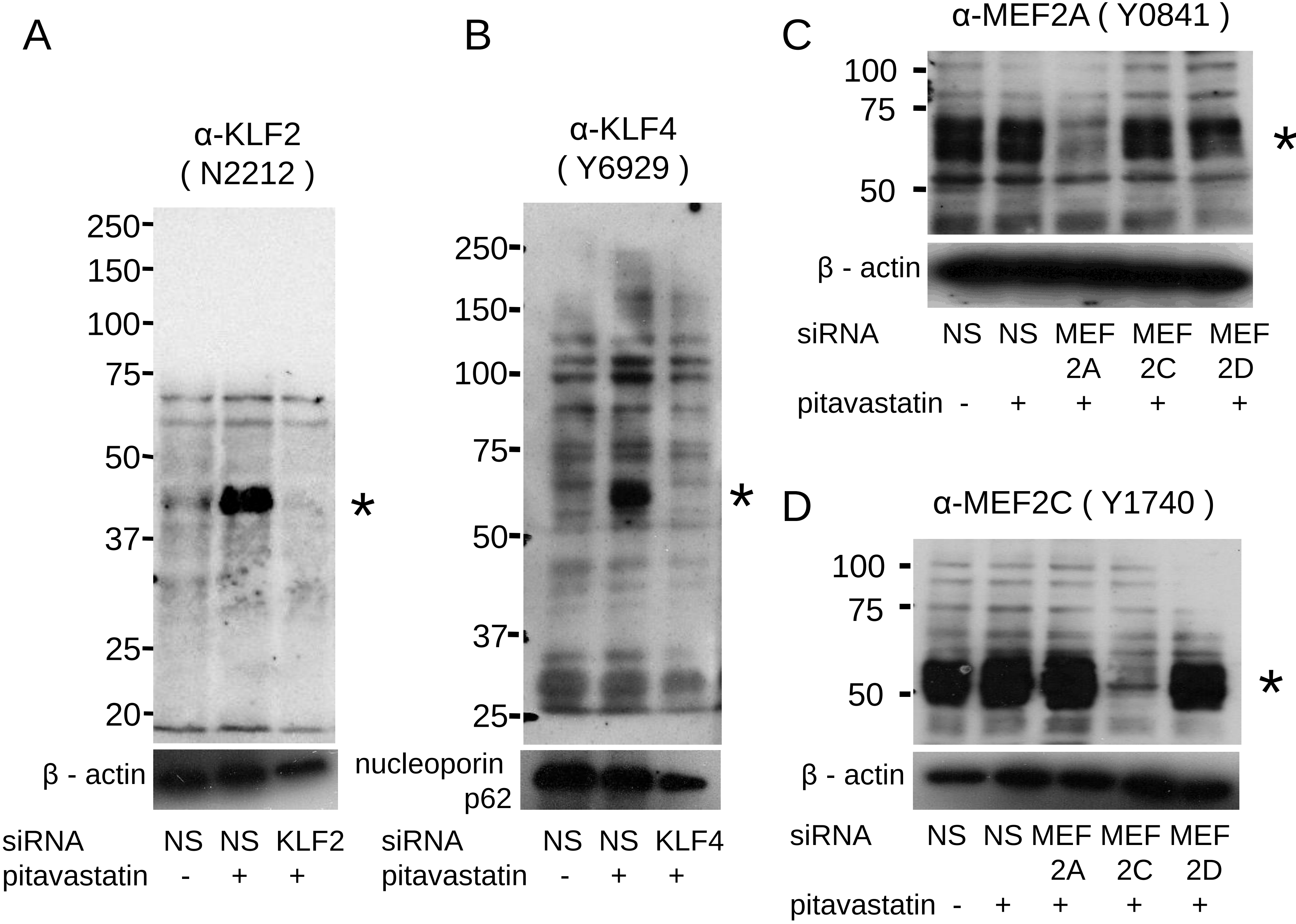

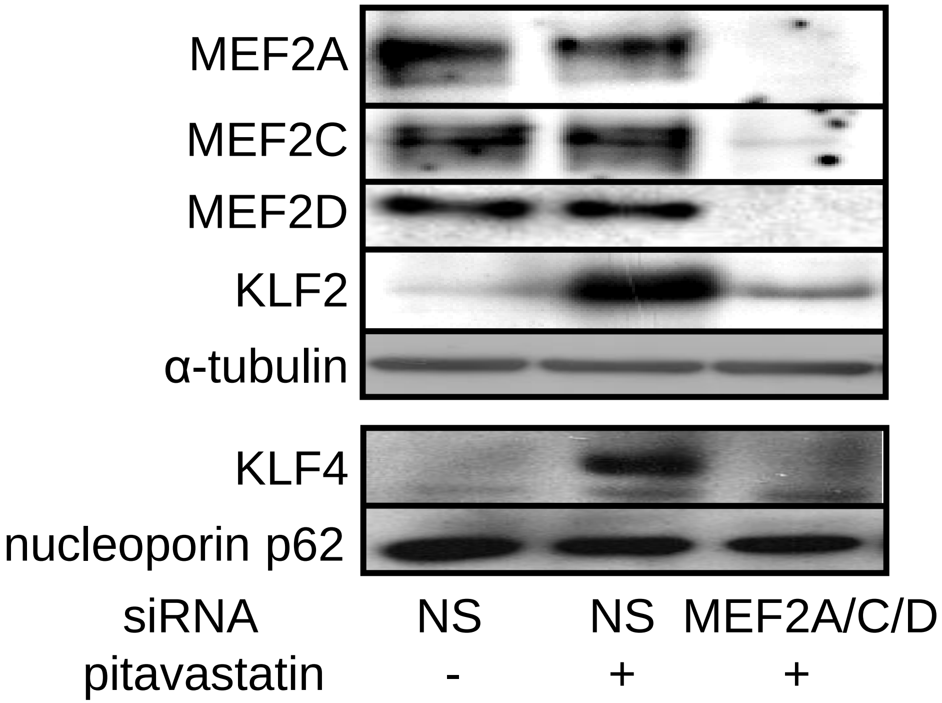
MEF2A
MEF2C
MEF2D
KLF2
α-tubulin
KLF4
nucleoporin p62
siRNA NS NS MEF2A/C/D
pitavastatin - + +

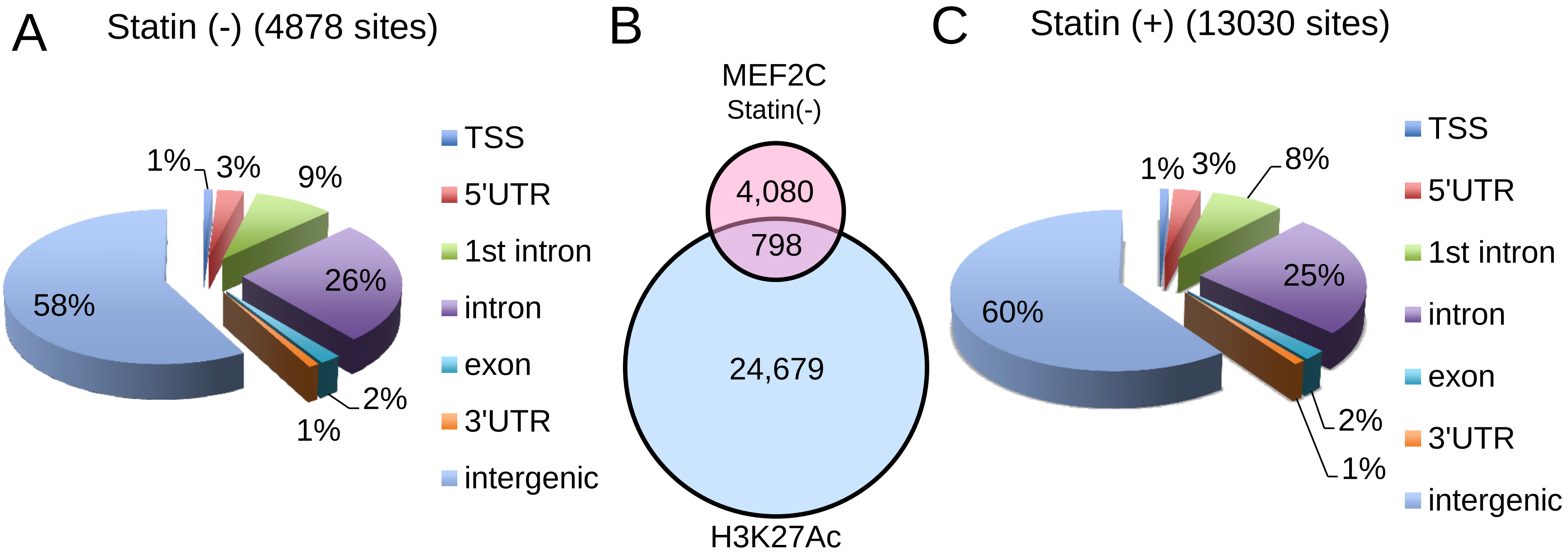
A
Statin (-) (4878 sites)
1%
3%
9%
26%
58%
2%
1%
TSS
5'UTR
1st intron
intron
exon
3'UTR
intergenic
B
MEF2C
Statin(-)
4,080
798
24,679
H3K27Ac
C
Statin (+) (13030 sites)
1%
3%
8%
25%
60%
2%
1%
TSS
5'UTR
1st intron
intron
exon
3'UTR
intergenic

A

peptide	phosphorylation	
C	none	SYSPTSPSYSPTSPSYSPC
Pa	S5	SYSPTSPSYSPTSPSYSPC
Pb	S7	SYSPTSPSYSPTSPSYSPC
Pc	S2	SYSPTSPSYSPTSPSYSPC
Pd	S5, S7	SYSPTSPSYSPTSPSYSPC
Pe	S5, S2	SYSPTSPSYSPTSPSYSPC
Pf	S7, S2	SYSPTSPSYSPTSPSYSPC
Pg	S7, S5	SYSPTSPSYSPTSPSYSPC
Ph	S2, S5	SYSPTSPSYSPTSPSYSPC
Pi	S2, S7	SYSPTSPSYSPTSPSYSPC

B

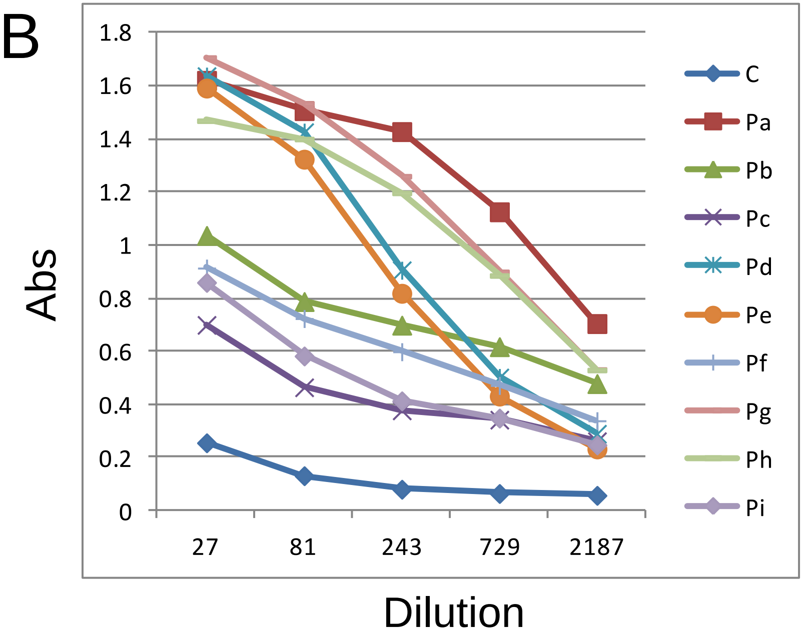

TGCAGCTTTCACAAGGTGGCAAAGCTTCTTCCAGTGTTGAATCTTTCTCT

KLF4 TSS **Hind III site** **-148 kb enhancer**

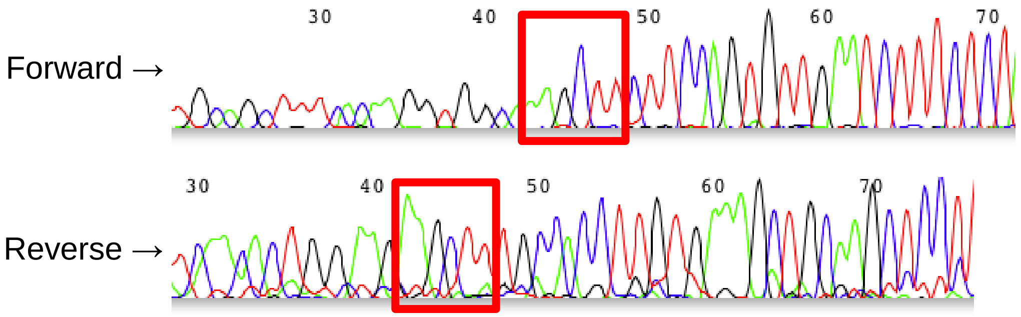

-148 kb enhancer **Hind III site** **KLF4 TSS**

CAACACTGGAAGAAGCTTTGCCACCTTGTGAAAGCTGCAAGCTCCT

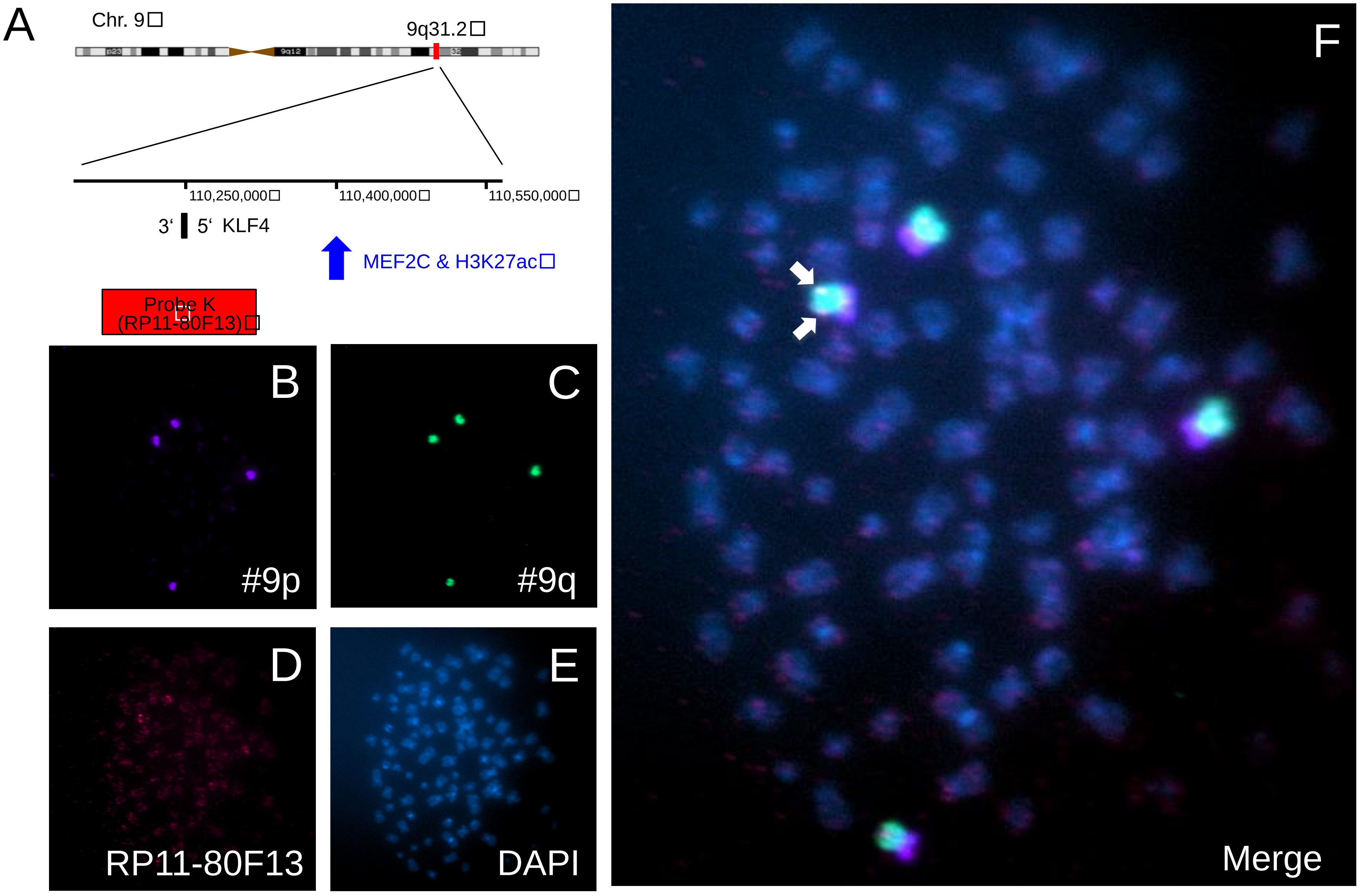
A
Chr. 9
9q31.2
p23
9q12
32
110,250,000
110,400,000
110,550,000
3‘ 5‘ KLF4
MEF2C & H3K27ac
Probe K
(RP11-80F13)
B
#9p
C
#9q
D
RP11-80F13
E
DAPI
F
Merge

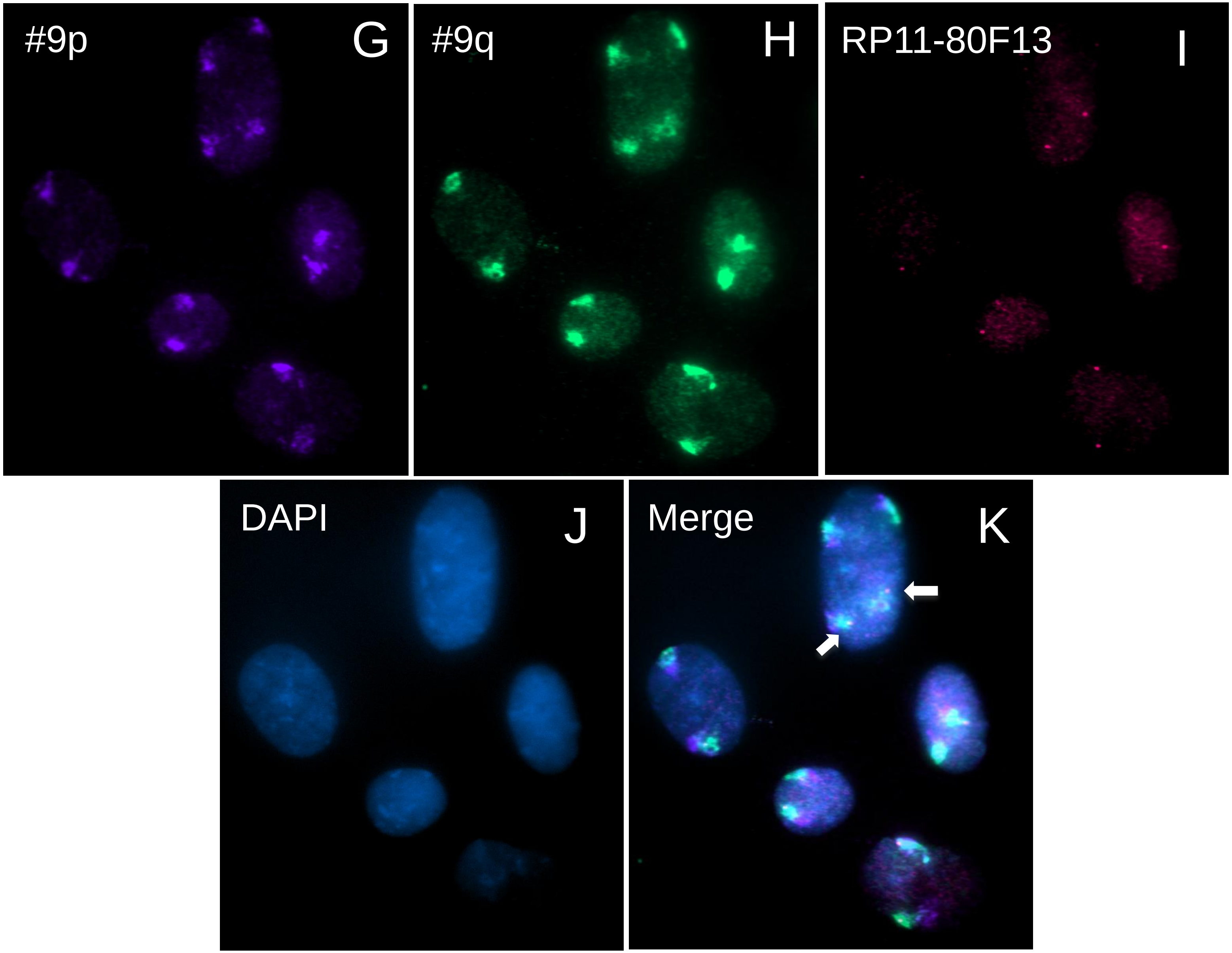
#9p
G
#9q
H
RP11-80F13
I
DAPI
J
Merge
K

Maejima&Inoue_Fig. S7

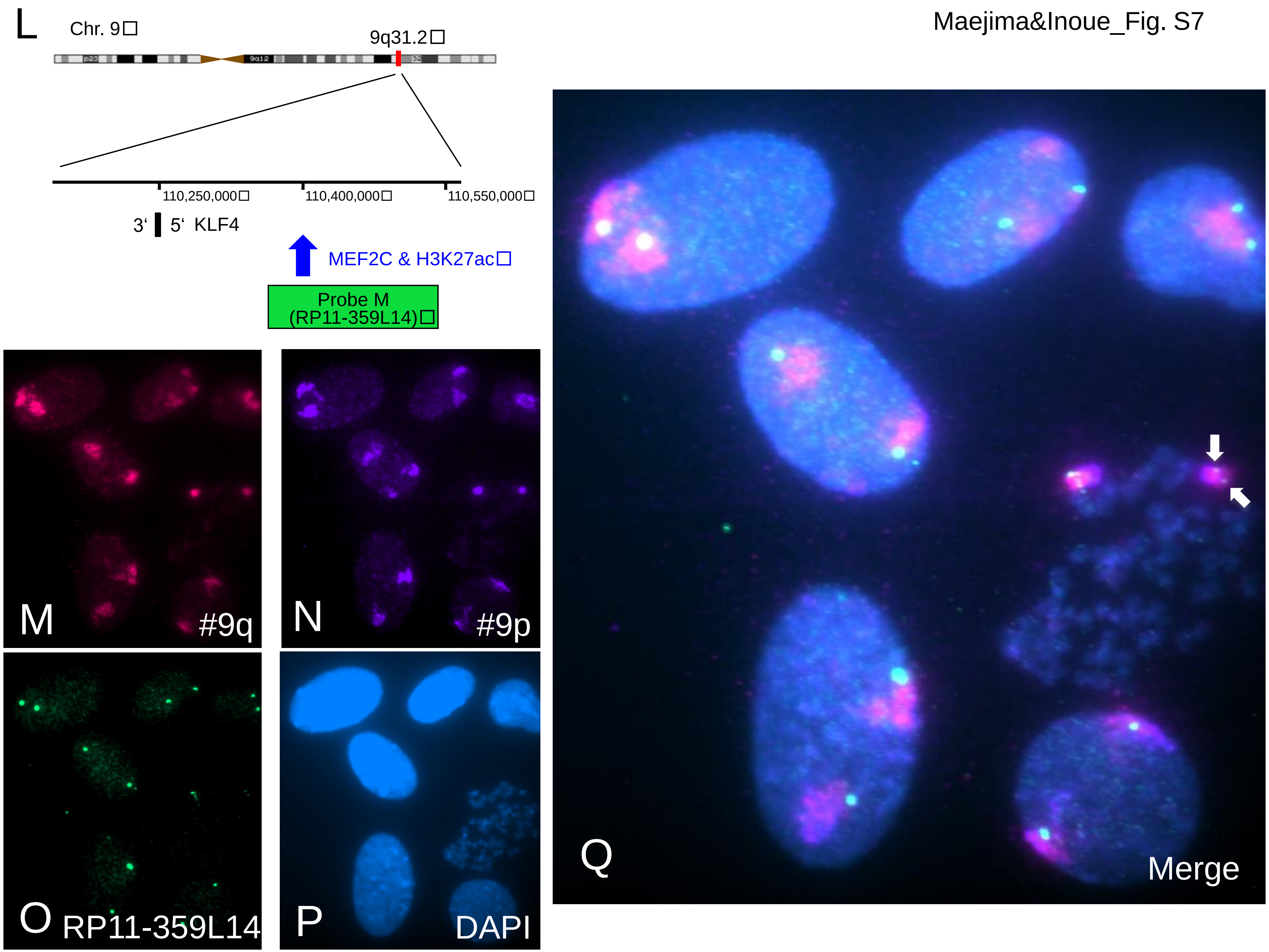

Table S1. List of genes induced by pitavastatin (top 20 genes)

Probe ID	Entrez Gene	Gene Symbol	Microarray analysis (Fold change compared to DMSO)
220266_s_at, 221841_s_at	9314	KLF4	13.03
219371_s_at, 226645_at, 226646_at	10365	KLF2	3.95
222162_s_at, 222486_s_at	9510	ADAMTS1	3.86
203887_s_at, 203888_at, 237252_at	7056	THBD	3.72
205749_at	1543	CYP1A1	3.09
230746_s_at	100288985	LOC100288985	2.58
208937_s_at	3397	ID1	2.34
203665_at	3162	HMOX1	2.31
206049_at	6403	SELP	2.24
229873_at	283219	KCTD21	2.21
207001_x_at, 208763_s_at, 235364_at	1831	TSC22D3	2.19
204284_at, 240187_at	5507	PPP1R3C	2.16
202393_s_at	7071	KLF10	2.12
214218_s_at, 221728_x_at, 224588_at, 224589_at, 224590_at, 227671_at, 235446_at, 243712_at	7503	XIST	2.11
215506_s_at	9077	DIRAS3	2.08
221087_s_at	80833	APOL3	2.00
206320_s_at, 227719_at	4093	SMAD9	1.99
225954_s_at, 231072_at	90007	MIDN	1.98
205581_s_at	4846	NOS3	1.98
229172_at, 234610_at	116835	HSPA12B	1.96

Table S2A. si RNA

MEF2A	AAUAAUCAGUGUUGUAGGCAGUCGG
MEF2C	UAAACCCAGACAGAGAUGACAGGUC
MEF2D	ACUAAAGGCUGGUAAGGAGGAGAGC
KLF4	AUUGGAGAGAAUAAAGUCCAGGUCC

Table S2B. Primer pairs for real Time PCR

Cyclophilin	Fw	TTCGTGCTCTGAGCACTGGAGA
	Rv	GGACCCGTATGCTTTAGGATGAAG
KLF4	Fw	GACGGCTGTGGATGGAAATTC
	Rv	CGGTGCCCCGTGTGTTTAC
NOS3	Fw	CCCTTCAGTGGCTGGTACAT
	Rv	TATCCAGGTCCATGCAGACA
Thrombomodulin	Fw	TTGCGAGAAGAGACAAACACCTC
	Rv	CAAGCTCCCAATTCCACAAGAC

Table S2C. Primer pairs for ChIP-PCR

KLF4 (-98 kb)	Fw	TTACCCAGCAAATCGGGAAGG
	Rv	AGCCGGGCTTCTGTGTGTAT
KLF4 (-148 kb)	Fw	GCTGCCTGACGTAGGGAGATA
	Rv	GGCCTCGGGAACTGACCA
KLF4 (-147 kb)	Fw	CAGGCAGTGCTAGGGCGTA
	Rv	GGGAATCTGGGAGGCTCAG

Table S2D. Primer pairs for reporter assay

For cloning gene		
KLF4 promoter	Fw	CAGGAGGCGGAGGTTGCACTGAG
	Rv	TAATGTGGGGGCCCAGAA
KLF4 enhancer (-98 kb)	Fw	TACCCAGCAAATCGGGAAGGAACTG
	Rv	GTTCTTTGGCGAACAACCTGCTCCG
KLF4 enhancer (-148 kb)	Fw	TGCGCTTCCCTCCGACGCGCGGAG
	Rv	TGCCTCGGGCCGGTCTTGCTCGG

Table S2E. Primers and TaqMan probes for 3C assay

KLF4 Fw	ACCCGAATTGCTTTGAAATGAA
KLF4 TaqMan probe	(6-FAM)TCCCTGCTAATAAATAAC(MGB)
KLF4 (-1kb) Rv	TCCAACCTGGGCAATAGAATG
KLF4 (-45 kb) Rv	TCTGTGACTCCAGCACCCAAG
KLF4 (-98 kb) Rv	TGCAACCCTGGCCAAGA
KLF4 (-120 kb) Rv	TTGTACTGGTTCATCACGGATTG
KLF4 (-148 kb) Rv	TGCGCACATACACACCATACTC
KLF4 (-180 kb) Rv	AGAAATACATCTCTCCTCAGACTAACTCTGA
